# Identification of Novel Molecular Subgroups in Esophageal Adenocarcinoma to Predict Response to Neo-Adjuvant Therapies

**DOI:** 10.3390/cancers14184498

**Published:** 2022-09-16

**Authors:** Sanne J. M. Hoefnagel, Willem J. Koemans, Hina N. Khan, Jan Koster, Sybren L. Meijer, Jolanda M. van Dieren, Liudmila L. Kodach, Johanna W. van Sandick, Silvia Calpe, Carmen M. del Sancho-Serra, Ana C. P. Correia, Mark I. Van Berge Henegouwen, Suzanne S. Gisbertz, Maarten C. C. M. Hulshof, Sandro Mattioli, Manon C. W. Spaander, Kausilia K. Krishnadath

**Affiliations:** 1 Center for Experimental and Molecular Medicine, Amsterdam UMC, 1105 AZ Amsterdam, The Netherlands; 2 Department of Gastroenterology and Hepatology, Amsterdam UMC, 1081 HZ Amsterdam, The Netherlands; 3 Cancer Center Amsterdam, 1081 HV Amsterdam, The Netherlands; 4 Department of Surgical Oncology, The Netherlands Cancer Institute, 1066 CX Amsterdam, The Netherlands; 5 Department of Pathology, Amsterdam UMC, 1066 CX Amsterdam, The Netherlands; 6 Department of Gastrointestinal Oncology, The Netherlands Cancer Institute, 1066 CX Amsterdam, The Netherlands; 7 Department of Pathology, The Netherlands Cancer Institute, 1066 CX Amsterdam, The Netherlands; 8 Esophageal Cancer Workgroup, Amsterdam UMC, 1081 HZ Amsterdam, The Netherlands; 9 Department of Surgery, University of Bologna, 40138 Bologna, Italy; 10 Department of Gastroenterology and Hepatology, Erasmus University MC, 3015 GD Rotterdam, The Netherlands; 11 Laboratory of Experimental Medicine and Paediatrics, Department of Gastroenterology and Hepatology, University Hospital Antwerp, University of Antwerp, 2650 Edegem, Belgium

**Keywords:** esophageal adenocarcinoma, RNA sequencing, subgroups, predicting response to therapy

## Abstract

**Simple Summary:**

Gene expression of esophageal adenocarcinoma is highly heterogeneous. In general, these cancers have poor prognosis and unpredictable responses to chemo- and radiotherapy. Investigating expression profiles from RNA from pre-treatment biopsies are highly attractive to investigate the existence of diverse biological groups and signatures associated with the clinical response to current treatment strategies. We identified and validated three distinct biological esophageal adenocarcinoma subgroups and identified immune signatures with association to therapy response using RNA sequencing. These findings aid in understanding biological mechanisms’ underlying response to neo-adjuvant treatment.

**Abstract:**

Esophageal adenocarcinoma (EAC) is a highly aggressive cancer and its response to chemo- and radiotherapy is unpredictable. EACs are highly heterogeneous at the molecular level. The aim of this study was to perform gene expression analysis of EACs to identify distinct molecular subgroups and to investigate expression signatures in relation to treatment response. In this prospective observational study, RNA sequencing was performed on pre-treatment endoscopic EAC biopsies from a discovery cohort included between 2012 and 2017 in one Dutch Academic Center. Four additional cohorts were analyzed for validation purposes. Unsupervised clustering was performed on 107 patients to identify biological EAC subgroups. Specific cell signaling profiles were identified and evaluated with respect to predicting response to neo-adjuvant chemo(radio)therapy. We identified and validated three distinct biological EAC subgroups, characterized by (1) p38 MAPK/Toll-like receptor signaling; (2) activated immune system; and (3) impaired cell adhesion. Subgroup 1 was associated with poor response to chemo-radiotherapy. Moreover, an immune signature with activated T-cell signaling, and increased number of activated CD4 T memory cells, neutrophils and dendritic cells, and decreased M1 and M2 macrophages and plasma cells, was associated with complete histopathological response. This study provides a novel molecular classification for EACs. EAC subgroup 1 proved to be more therapy-resistant, while immune signaling was increased in patients with complete response to chemo-radiotherapy. Our findings give insight into the biology of EACs and in cellular signaling mechanisms underlying response to neo-adjuvant treatment. Future implementation of this classification will improve patient stratification and enhance the development of targeted therapies.

## 1. Introduction

Esophageal and esophageal junctional adenocarcinomas (EAC) are highly aggressive, with five-year overall survival rates that rarely exceed 20% [1]. Neoadjuvant chemoradiotherapy (nCRT) followed by esophagectomy is considered standard of care in many centers for patients with locally advanced esophageal or junctional cancer [2]. The response of EAC to nCRT is highly variable and unpredictable. Advances in therapy have achieved incremental improvements in overall outcome, but over- and under-treatment of undefined subgroups of patients might undermine these benefits [3]. For these reasons, there are still centers that advocate only surgical resection with extensive two-field lymph node dissection of EAC patients [4]. The biological diversity of EACs complicates patient selection and treatment stratification and impedes the development of new targeted agents. To date, beneficial evidence of targeted therapies for locally advanced esophageal or junctional cancer is lacking, despite emerging targeted therapies for many types of cancer. Attempts to develop prognostic tools for therapy response, including serum [5] and pathological markers [6] have been made, but suboptimal accuracy and lack of validation in independent patient cohorts limit clinical implementation. Further insight in the heterogeneous molecular pathology of EAC and its relation to response to current treatment strategies is needed.

Over the years, molecular characterization using genomic, transcriptomic, epigenomic and proteomic platforms, has been performed for many types of cancer, including esophageal and gastric adenocarcinoma and esophageal squamous cell carcinoma.

Whole-genome sequencing highlights the highly diverse mutational landscape of EAC. Dulak et al. have shown that EAC has a high overall mutation frequency, only exceeded in lung cancer [7]. By cluster analysis based on mutational signatures obtained by WGS in another patient cohort, six mutational signatures were seen to some extent in most patient tumors, underlining heterogeneity in the mutational spectrum. Three distinct molecular subgroups (1. c>A/T dominant, 2. DNA damage repair (DDR) impaired, 3. increased mutagenic signature) were identified by assigning patients to a specific subgroup according to their most dominant mutational signature. Unfortunately, the prognostic value of these genomic-based clusters is limited, as no correlation with clinical characteristics, such as response to chemotherapy and overall or recurrence-free survival, was found [8].

Most genomic sequencing approaches consider the malignant epithelial cells, but not the stromal compartment of the tumor and potential epithelial-mesenchymal crosstalk. However, the stromal compartment might have intrinsic prognostic value, as shown in pancreatic cancer with separate tumor- and stroma-specific subgroups with prognostic relevance [9]. Analyzing gene expression from bulk tumors with contribution from epithelial cancer cells and stromal cells may overcome this limitation. Another advantage of gene expression analysis over other platforms is that this single platform is able to capture the most important features identified by comprehensive multi-platform molecular characterization, as shown for gastric adenocarcinoma [10]. Expression data was shown to recapitulate the most important features of the final, comprehensive, molecular characterization, underlining the potential of gene expression-based clustering to lead to robust subgrouping.

At present, gene expression profiles generated by RNA sequencing on treatment-naïve material of patients with EAC, which were subsequently treated by chemoradiotherapy (CRT), are not available. The molecular profiles from large publicly available RNA-seq databases, including The Cancer Genome Atlas (TCGA) database, have been mostly established from tissues obtained from surgical resection specimens of patients, who were treated by surgery alone and thus cannot be extrapolated to predict patient response to neo-adjuvant therapies [11].

Gene expression profile-based classification has the potential to identify tumor- and stromal-intrinsic subclasses to further elucidate molecular heterogeneity and potential association with treatment response. More insight into specifically dysregulated signaling pathways in subgroups of EAC patients may facilitate patient stratification for, and development of therapies targeting, these specific pathways.

In this study, we developed a robust molecular classification for EACs with association to clinical characteristics. We identified dysregulated pathways and candidate drivers of distinct subgroups of EACs that could be targeted therapeutically. Moreover, we found a differentially expressed immune phenotype between patients with different pathological responses to CRT [12]. Independent cohorts of EAC patients were used for validation of our findings.

## 2. Materials and Methods

### 2.1. Study Population and Tissue Samples

A total of 110 patients with primary EAC from the Amsterdam University Medical Center (AUMC) in Amsterdam (referred to as discovery cohort) were included. Tissue samples were collected with approval of the local medical ethics committee and all patients provided written informed consent (AMC2013_241).

All patients underwent esophago-gastro-duodenoscopy and surgery with or without neo-adjuvant treatment between 2012 and 2017. During this procedure, biopsies from the tumor mass were obtained for diagnostic and research purposes. Histological diagnosis and Mandard classification on resection specimens were performed by specialized gastro-intestinal pathologists as part of standard clinical care. For the discovery cohort, blinded examination of biopsies to confirm histological diagnosis was performed by a specialized gastro-intestinal pathologist (SLM).

Only patients with adenocarcinoma of the esophagus and of the gastro-esophageal junction were included. Definition of the location was retrieved from the description of the location in the endoscopy report. Siewert classification was not routinely performed [13].

Pre-treatment tumor staging was performed by esophago-gastro-duodenoscopy, endoscopic ultrasound and CT scan. AJCC staging was described according to the 8th edition of the UICC TNM-classification. Only patients with T2 to T4 stage were included in this study. In 10 patients, biopsies from adjacent normal esophageal mucosa were taken at least 3 cm from the tumor mass. The biopsies were immediately immersed in RNAlater solution and stored at −80 degrees until further processing. Data on clinical, radiological and pathological characteristics were retrospectively extracted from the electronic medical records.

### 2.2. Cluster Analyses

RNA preparation, expression profiling, normalization and estimation of tumor percentage in the discovery cohort was performed as described in “RNA preparation discovery cohort”, “RNA seq expression profiling discovery cohort”, and “Bioinformatics analyses” in the Supplementary File S1 methods. Unsupervised clustering analysis of 107 gene expression profiles from EAC was performed using the ConsensusClusterPlus function with hierarchical cluster algorithm with Euclidean distance function, based on gene median-centered expression values of 16,045 genes [14]. Details from this analysis are described in “ConsensusClusterPlus” in the Supplementary File S1 methods.

All samples were plotted on Principal Component Analysis plots.

Differential expression analyses and gene set enrichment analyses for subgroups from the discovery cohort was performed as described in “Differential expression analyses and Ingenuity Pathway Analysis” in the Supplementary File S1 methods.

Using the discovery dataset, a random forest model was trained as described in “Random forest model” in the Supplementary File S1 methods.

The random forest model that was trained in the discovery cohort was applied to predict EAC subgroups in the EAC samples from the TCGA database after normalization similar as described for the discovery cohort and batch effect correction. ISOpureR analysis was performed on TCGA expression profiles of protein-coding genes from 80 EAC samples and 11 corresponding healthy tissue samples, and only TCGA samples with an estimated tumor percentage of 50% or higher (n = 77) were used in analyses.

Differential expression analysis and IPA were performed for the TCGA dataset similarly as described for the discovery cohort.

Heatmaps were used for visualization of differentially expressed genes and significantly enriched pathways, as described in “Visualization of differentially expressed genes and significantly enriched pathways” in the Supplementary File S1 methods.

For the TCGA dataset, mutation data was retrieved and analyzed as described in “mutation data from the TCGA dataset” in the Supplementary File S1 methods.

### 2.3. CIBERSORTx

We used CIBERSORTx to estimate the absolute quantities of specific cell types from the bulk tissue gene expression profiles from the discovery and the RNA sequencing validation cohorts [15]. In short, CIBERSORTx estimates absolute immune fraction score by dividing the median expression level of all genes in the signature expression matrix by the median expression of all genes in the bulk tissue expression matrix. CIBERSORTx is a machine-learning method for digital cytometry, which enables inferring cell-type-specific gene expression profiles from bulk tissue transcriptomes [16]. The CIBERSORTx analysis was run using LM22 (22 immune cell types) as the signature gene file, with 100 permutations and quantile normalization disabled as recommended for RNA-Seq data.

### 2.4. Validation of the three Biological Subgroups of EAC Patients Treated by Surgery Only Using Nanostring Technology

For validation of the clusters, expression profiles produced by Nanostring technology were obtained from an independent cohort of patients treated by surgery only. MRNA was isolated from macro-dissected formalin-fixed paraffin-embedded (FFPE) chemo- and radiotherapy naïve surgical resection specimens using the RNeasy FFPE kit (Qiagen, Germantown, MD, USA, Cat. No. 73504). Samples with good RNA quality, defined by Bioanalyzer (Agilent, Santa Clara, CA, USA), were used for expression profiling by the Nanostring PanCancer Progression panel (nanoString, Seattle, WA, USA, XT-CSO-PROG1-12). The obtained expression profiles were presented to the random forest model to assign cluster membership.

### 2.5. Supervised “Response to Chemoradiotherapy According to CROSS” Analyses in the Discovery Cohort

After unsupervised cluster analysis, we performed two supervised analyses by dividing patients from the discovery cohort treated with nCRT and neo-adjuvant chemotherapy (nCT) in 4 subgroups according to, respectively, tumor regression grade as defined by Mandard [12] and pathological T-stage (pT) as seen in the resection specimen.

In the first analysis, we compared (A) patients with Mandard 1 versus patients with Mandard 2, 3, 4 and 5, (B) patients with Mandard 1 and 2 versus Mandard 3, 4 and 5, and C) patients with Mandard 1, 2, and 3 versus patients with Mandard 4 and 5 using differential expression analysis genes by DESeq2.

In the second analysis, we compared (I) patients with pTx versus patients with pT1, pT2, pT3, (II) patients with pTx and pT1 versus patients with pT2 and pT3, and III) patients with pTx, pT1 and pT2 versus patients with pT4. Differentially expressed genes (*p* < 0.05) were visualized in MA plots after log fold change shrinkage.

Gene set enrichment analyses (GSEA) was performed to investigate if there was significant enrichment (nominal *p*-value < 0.05) of KEGG pathways [17,18] between patients grouped by Mandard score. Normalized enrichment scores of significantly enriched pathways were visualized in heatmaps.

The same analysis was performed for patients treated with nCRT according to CROSS and surgical resection, with or without adjuvant therapy, and available pT and Mandard score data and results are shown in the Supplementary Figures.

Validation cohorts containing 51 samples from patients with EAC treated with nCRT according to the CROSS regimen, followed by surgery from the NKI and Erasmus MC, were sequenced as described in “NKI/Erasmus MC RNA seq validation cohort for response to neo-adjuvant treatment signature” in the Supplementary File S1 methods. CIBERSORTx data was compared between patients with different Mandard scores in the discovery cohort and in the two validation cohorts from the NKI and Erasmus MC.

### 2.6. Statistics Patients Characteristics

Patient characteristics from the discovery cohort, the TCGA cohort, the Bologna/AUMC surgery-only cohort and the NKI/Erasmus MC cohort were compared using the Fisher-Freeman-Halton Exact test (2-sided), the One-Way ANOVA test, and the Chi-square test, to test for clinical differences between subgroups for, respectively, binary variables, not normally distributed continuous variables, and categorical variables. Statistical significance was set at a *p* value of <0.05.

Kaplan-Meier survival analyses were performed to compare survival. River plots were used for visualization of cT and pT before and after nCRT [19].

## 3. Results

### 3.1. EAC Patients from One Dutch Academic Center Were Included as the Discovery Cohort and Patients with EAC from the TCGA Dataset Served as the Validation Cohort for Cluster Analysis

The RNA-seq discovery cohort consisted of 107 Dutch patients from the AUMC with histologically proven primary EAC (median age 66.7 years, IQR 15.7) (Table 1). 77 patients with EAC from the TCGA dataset were used as an independent cluster validation dataset (median age 68.0 years, IQR 19.0) (Table 1).

There were no differences between the discovery and TCGA cohorts regarding male/female ratio (*p* = 0.65) and age (*p* = 0.56) (Supplementary Table S1). The histological grade was significantly different between patients in the discovery and the TCGA database (*p* = 0.00) (Supplementary Table S1).

Patients from the discovery cohort were diagnosed between 2011 and 2017.

68 out of 107 patients received nCT or nCRT before surgery, and of these 46 were treated with CROSS followed by surgery (Table 1). 23 out of 107 patients underwent nCRT without surgery or definitive chemoradiotherapy (dCRT). Patients from the TCGA cohort were diagnosed between 1998 and 2013, and none of the patients in the TCGA cohort were treated with nCRT prior to surgery.

AJCC stage at baseline and clinical T stage (cT) at baseline were missing for >50% of cases from the TCGA, therefore statistical comparison was not performed for these variables.

Finally, a total of 107 patients from one academic center, mostly treated with neo-adjuvant therapy and surgery, were included as the discovery cohort, and patients with EAC from the TCGA dataset, mostly treated with surgery only, were used for the validation analyses.

### 3.2. Unsupervised Cluster Analysis by Consensus Cluster Plus in the Discovery Cohort and Validation in the TCGA Cohort

**Figure 1:**
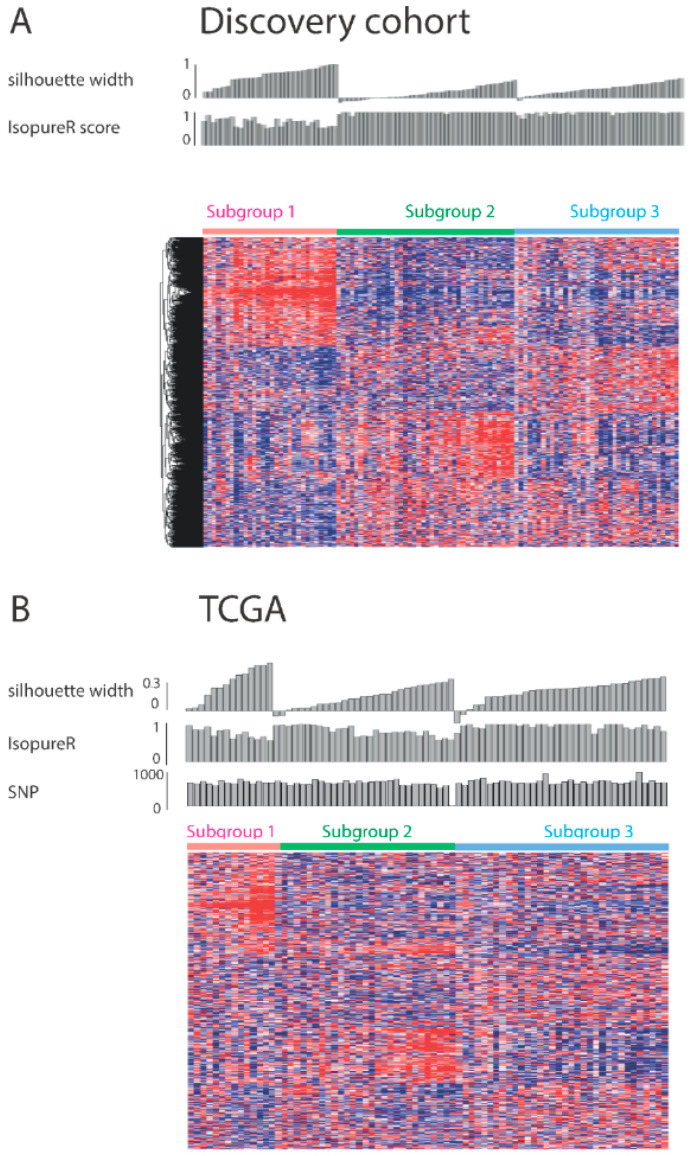
Heatmaps indicating expression levels of the AUMC and TCGA cohorts. Rows with differentially expressed genes ordered by unsupervised clustering in AUMC cohort are shown by the dendrogram at the left of the AUMC heatmap. Columns with EAC RNA-seq profile for each patient are ordered by silhouette width and cluster membership.

Data-driven, unsupervised consensus clustering analysis was performed to identify subgroups. This analysis identified three stable subgroups in the discovery cohort. The Consensus matrix for k = 3 showed that most patient samples could be assigned uniquely to the same subgroup (Supplementary Figure S1A). The optimal separation in three groups was corroborated by the cumulative distribution function (CDF) (Supplementary Figure S1B), and by delta area plot visualization (Supplementary Figure S1C) [14]. The Principal Component Analysis (PCA) plot (Supplementary Figure S1D) underscored that also after data reduction, the three distinct subgroups could still be recognized. Silhouette width plots for the discovery dataset confirmed that most samples assigned to a specific subgroup were far away from the decision boundary between neighbouring clusters (Figure 1).

By applying random forest modelling, similar subgroups could be identified in the TCGA cohort. Principal component analysis confirmed the existence of these subgroups (Supplementary Figure S2). Silhouette width plots for the TCGA dataset confirmed that most samples assigned to a specific subgroup were far away from the decision boundary between neighbouring clusters (Figure 1).

In conclusion, three distinct stable subgroups could be identified in the discovery cohort by cluster analysis, and could be validated in the TCGA cohort by random forest modelling.

### 3.3. Genomic Data from the TCGA Dataset Indicates Similar Mutational Loads between the Subgroups as Defined by the RNA Expression Profiles

To investigate the genomic composition of the three subgroups, we used public data from the TCGA cohort. The patients from the different subgroups of the TCGA cohort proved to have a similar amount of mutations (*p* = 0.13 according to Kruskal-Wallis rank sum test) and copy number variations (*p* = 0.25 according to one-way ANOVA for amplifications, *p* = 0.88 according to Kruskal-Wallis rank sum test for deletions, *p* = 0.218 according to one-way ANOVA for amplifications and deletions) (Figure 1). There were no specific genes with significantly different frequencies of SNV mutations or copy number variations between the subgroups.

Analysis of genomic data indicates that the subgroups based on gene expression profiles were not associated with microsatellite instability or other baseline genomic abnormalities, including p53 gene mutations.

### 3.4. Clinical and Histo-Pathological Characteristics of the Subgroups

We examined whether there were differences in clinical and pathological characteristics among the three subgroups. In the discovery cohort, there was no significant difference in age, male/female ratio or AJCC stage between the three groups (age *p* = 0.47, sex *p* = 1.00, AJCC stage *p* = 0.80) (Table 2). In the TCGA cohort, there were also no significant differences in age nor in male/female ratio between the subgroups (age *p*= 0.28, sex *p* = 1.00) (Supplementary Table S1).

There were significant differences between histologic grade between the discovery cohort and the TCGA dataset (Table 1), and also between subgroups in the discovery cohort and in the TCGA cohort (Table 2, Supplementary Table S1).

For both cohorts there was a similar tendency for better overall survival for subgroup three (Supplementary Figure S3A,B).

In conclusion, histological grades were different between the subgroups in both the discovery cohort and in the TCGA cohort. There were no significant differences in age, male/female ratio or survival.

### 3.5. Differential Gene Expression and Pathway Enrichment Analyses Identifies Specific (Aberrant) Signaling Pathways within Each Subgroup

To identify specific signaling pathways within the subgroups, differential gene expression and pathway enrichment analyses were performed. 5720 genes were differential expressed when comparing subgroup one versus subgroups two and three, and, respectively, 5522 genes and 4325 genes when comparing subgroup two versus subgroups one and three, and subgroup three versus subgroups one and two in the discovery dataset.

**Figure 2:**
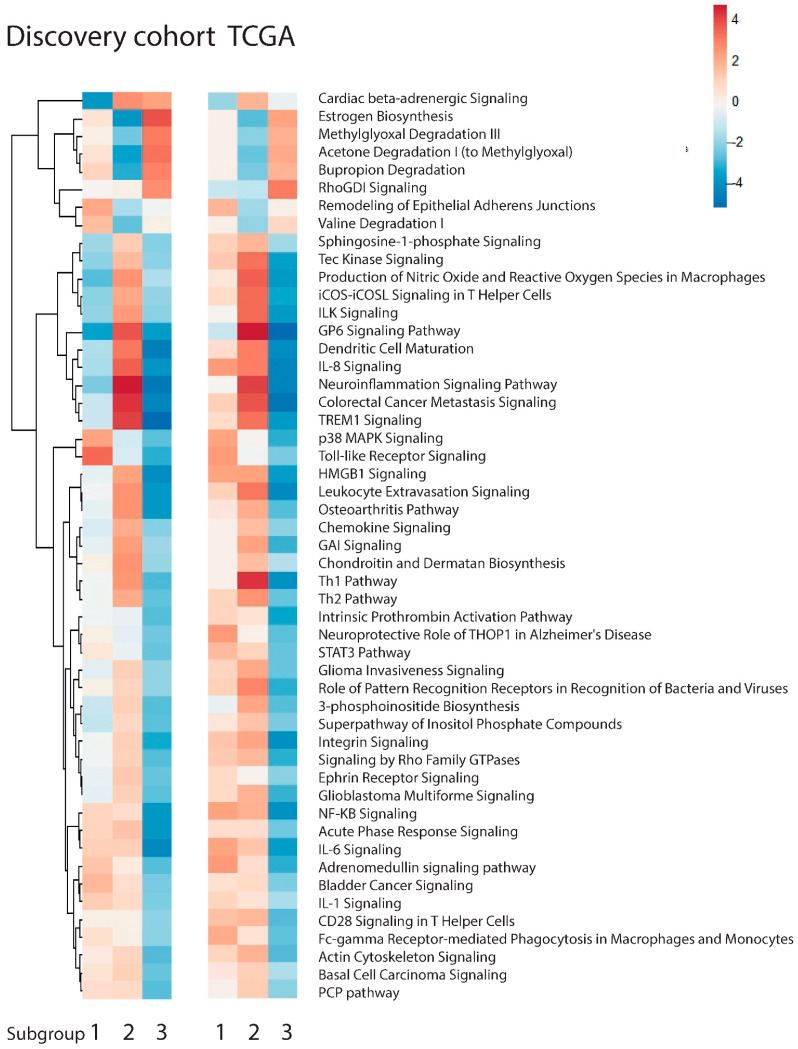
Heatmaps indicating IPA results for the AUMC (**left**) and TCGA (**right**) cohorts. In red, activated pathways, and in blue, de-activated pathways when comparing one subgroup to the other two subgroups.

In both the discovery and TCGA cohorts, differential-expression analysis and Ingenuity Pathway Analyses revealed specific expression signatures for each of the three subgroups (Figure 2). The groups could be classified as: 1. the p38 MAPK/Toll-like receptor signaling subgroup, 2. the activated immune system subgroup, and 3. the impaired cell adhesion subgroup.

Subgroup one was characterized by activation of the p38 MAPK signaling pathway, Toll-like Receptor signaling and remodelling of epithelial adherens junctions.

Subgroup two can be distinguished from the other subgroups because of the activation of immune pathways, including the Th1 pathway, dendritic cell maturation, leukocyte-extravasation signaling, and the production of nitric oxide and reactive oxygen species in macrophages. In addition, the colorectal cancer metastasis signaling pathway was activated in this subgroup, confirming altered expression of genes important for enhanced cancer cell migration, which potentially leads to metastatic cancer sites.

Subgroup three contained samples characterized by the deactivation of pathways involved in cell adhesion, such as integrin signaling, actin cytoskeleton signaling, and chondroitin and dermatan biosynthesis. Of great interest, several immune pathways were deactivated, including Th2 signaling, dendritic cell maturation, leukocyte extravasation signaling and CD28 signaling in T-helper cells. The only activated pathway in this subgroup was the RhoGDI signaling pathway.

In short, the activation of p38 MAPK and Toll-like receptor signaling in subgroup one, and of the pathways important for the immune system in subgroup two, were seen, whereas the impaired activation of pathways implied in cell adhesion was seen in subgroup three.

### 3.6. Validation of the Subgroups by Nanostring Technology on RNA from FFPE Samples

To validate the clustering results on FFPE samples by using a smaller panel of genes, the Nanostring PanCancer Progression panel (nanoString, XT-CSO-PROG1-12) was applied. This pre-defined 770-gene set, consisting of genes covering various steps in the oncogenesis process, proved to overlap with the significantly differentially expressed genes between the groups as identified in the discovery and validation cohorts (Supplementary Figure S4). FFPE samples from surgical resection specimens of an independent cohort of 56 patients, who did not receive adjuvant or neo-adjuvant therapy, but were treated by surgery only, were obtained from the AUMC (n = 26) and from the university hospital of Bologna (n = 30) (Supplementary Table S2). Patients had a median age of 71.0 years, IQR 12.0, and most patients were male (79%) (Supplementary Table S2). Around half of the patients had AJCC stage III disease at baseline (46%) (Supplementary Table S2). Gene expression profiles using the Nanostring technology on FFPE samples to assess the PanCancer progression panel gene set proved to be sufficient to distinguish between three subgroups in the mixed Bologna/AUMC cohort when applying the random forest model (Supplementary Figure S5A,B, Table S3).

In conclusion, these results indicate that the subset of genes as present in the Nanostring PanCancer panel applied on FFPE tissue is sufficient to identify EAC patients belonging to one of the subgroups.

### 3.7. CIBERSORTx to Investigate Differences in Immune Cells within the Three Subgroups

We found that subgroup two could be distinguished from the other subgroups because of the activation of immune pathways and activation of different types of immune cells. To further estimate the absolute quantities of specific immune cell types, we performed CIBERSORTx. This analysis confirmed that samples from subgroup two in both cohorts had a more active immune system compared to the other samples, with more activated CD4 memory T-cells (discovery *p* = 0.003 TCGA *p* = 0.026 according to MWU-test) and more resting mast cells (discovery *p* = 0.004 TCGA *p* = 0.009). CIBERSORTx analyses also confirmed impaired immune expression in subgroup three. Subgroups one and two together had more M1 (pro-inflammatory) macrophages (discovery *p* = 0.028 TCGA *p* = 0.010) than subgroup three (Supplementary Figure S6).

In conclusion, in both cohorts, increased numbers of immune cells were seen in subgroup two, and a decreased number of macrophages in subgroup three.

### 3.8. Response Prediction by Unsupervised Clustering of Patients Treated with Neoadjuvant Chemoradiotherapy Combined with Surgery

Since a subset of cases from the discovery cohort received neo-adjuvant chemo- or chemoradiotherapy before surgery, we next set out to investigate if we could identify an RNA signature associated with response to neo-adjuvant (chemo- with or without radiotherapy) treatment. For this sub-analysis, data was available for 65 patients from the discovery cohort.

**Figure 3:**
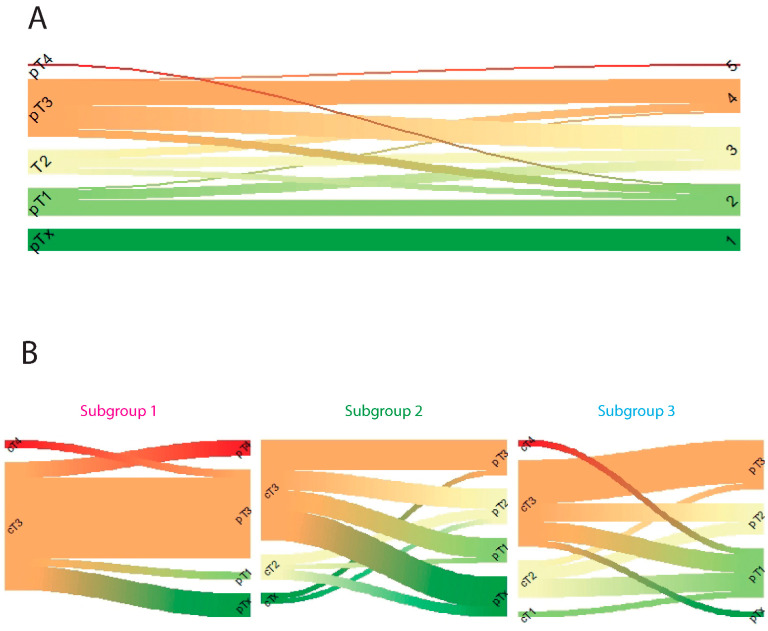
(**A**) pT stage and Mandard score (1, 2, 3, 4, 5) are highly correlated. (**B**) Clinical T stage and pathological T stage after neo-adjuvant treatment in 3 clusters in the AUMC cohort.

Two different response classifications to neo-adjuvant therapy were evaluated. Patients were classified according to the Mandard score and according to the pT (Figure 3A).

Analysis of therapy response between the different subgroups indicated that there was no difference for cT before treatment (*p* = 0.25, Table 2). The response to therapy depicted by Mandard score did not significantly differ between the subgroups (chi-square test *p* = 0.18, Table 2). However, for the pT there was a significant difference between subgroups (chi-square test *p* = 0.01, Table 2). Individually matched cT and pT scores for each subgroup shows that a large proportion of patients from subgroup one does not show pT downstaging upon treatment, compared to subgroups two and three (Figure 3B). This indicates that subgroup one is more resistant to neo-adjuvant therapy. For the subgroup of patients treated by nCRT according to CROSS (followed by surgical resection with and without adjuvant S-1 plus oxalipatin (SOX)) (pT and Mandard score available for n = 54 out of 56) a similar trend was seen for pT-staging (chi-square test *p* = 0.07, Supplementary Figure S7).

According to these results, the subgroups identified by unsupervised cluster analysis can potentially serve as an aid for predicting response to neo-adjuvant therapy. This classification shows less pT downstaging upon neo-adjuvant therapy in patients from subgroup one in the discovery cohort.

### 3.9. KEGG Pathway Analysis to Identify Specific Pathways in Responders versus Non-Responders to Neo-Adjuvant Therapy

**Figure 4:**
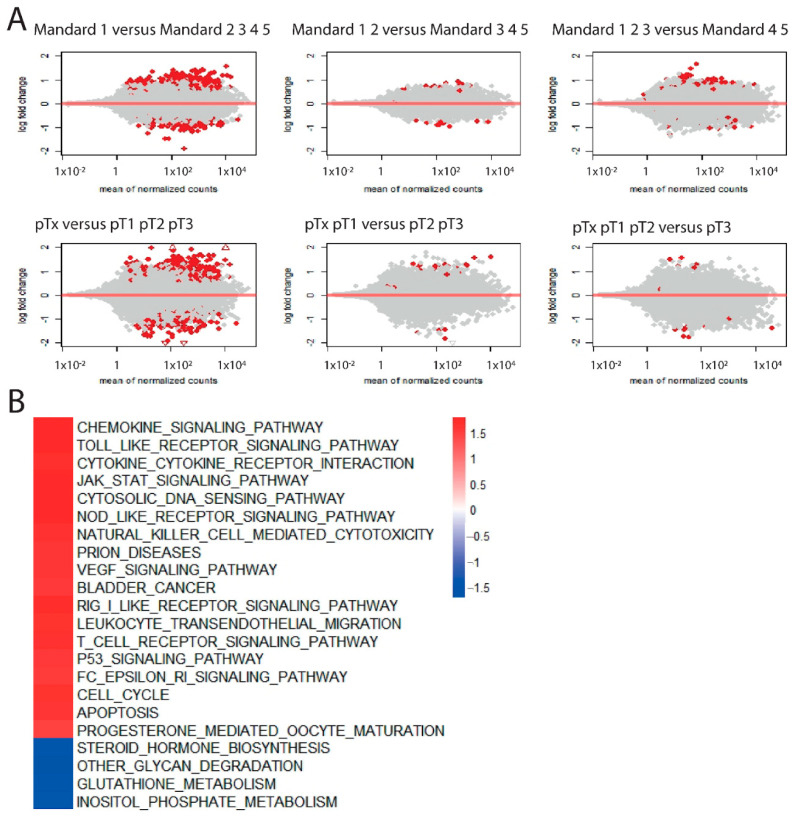
(**A**) MA plots indicating the results of supervised investigation by differential expression analyses in 65 patients treated with nCT/nCRT from the AUMC discovery cohort. Differentially expressed genes in red between patients with Mandard 1 versus higher Mandard scores (left upper plot) and pTx versus higher pT stadia (left lower plot). (**B**) Heatmap indicating GSEA results for patients treated with nCT/nCRT from the AUMC cohort. In red, enriched KEGG pathways in patients with Mandard 1, and in blue, enriched KEGG pathways in patients with higher Mandard scores.

At the gene expression level, the highest number of differentially expressed genes were between patients with complete response (Mandard 1 or pTx) versus patients with incomplete response (Mandard 2/3/4/5 or pT1-pT3) (Figure 4A). Analysis for Enriched KEGG pathways indicated that complete responders to neo-adjuvant therapy had increased signaling of several immune signaling pathways, including the T-cell receptor signaling pathway, leukocyte trans-endothelial migration and natural killer cell mediated cytotoxicity (Figure 4B). In addition, for patients treated by nCRT according to CROSS (followed by esophagectomy with and without adjuvant SOX), these immune pathways were significantly enriched in complete responders (Supplementary Figure S8).

In sum, patients with a complete response to neo-adjuvant therapy showed more activation of several immune signaling pathways compared to incomplete responders in the discovery cohort.

### 3.10. CIBERSORTx Shows a Specific Immune Phenotype in Complete Responders Compared to Incomplete Responders

In the discovery cohort, both differential gene expression analysis and CIBERSORTx indicated that the response to neo-adjuvant CRT was associated with a specific immune signature, expressed by the stromal and infiltrating immune cells. To confirm these findings, EAC patients with differential response to CRT obtained from the NKI (n = 29) and the Erasmus MC (n = 22) were analyzed and compared to the discovery cohort.

**Figure 5:**
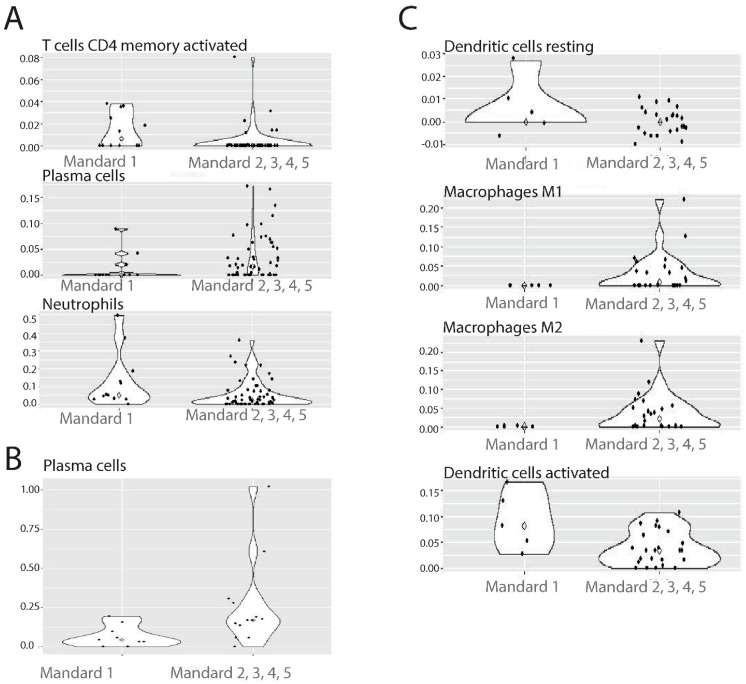
(**A**) CIBERSORTx Mandard Violin plots AUMC. (**B**) CIBERSORTx Mandard Violin plots Erasmus MC. (**C**) CIBERSORTx Mandard Violin plots NKI. Every dot depicts a sample. White diamond depicts median value.

CIBERSORTx analysis was used to compare patients with complete response (Mandard 1) to patients with incomplete response (Mandard 2, 3, 4, 5) with regard to their absolute quantities of specific immune cell types. Patients from the discovery cohort treated with nCT/nCRT and complete response had a significantly higher number of activated CD4 T memory cells (*p* = 0.004 according to MWU-test), and neutrophils (*p* = 0.036) (Figure 5A), while the number of plasma cells was estimated to be lower (*p* = 0.036) in the discovery cohort (Figure 5A). The number of plasma cells was also lower in complete responders (*p* = 0.019) from the Erasmus MC cohort (Figure 5B), while M1 and M2 Macrophages were lower in the complete responders from the NKI cohort (*p* = 0.043, *p* = 0.043) (Figure 5C). In the NKI cohort, patients with Mandard 1 had a higher number of both resting and activated dendritic cells (*p* = 0.036, *p* = 0.046) in comparison with incomplete responders (Figure 5C). From these results, it seems that distinguishing the number and types of infiltrating immune cells is associated with histopathological response to therapy. Good response to CRT seems to be associated with high numbers of activated CD4 T memory cells, neutrophils, and resting and activated dendritic cells, and low numbers of plasma cells and M1 and M2 macrophages.

Thus, increased numbers of several immune cells were found in patients with complete response to neo-adjuvant therapy, compared to incomplete responders in the discovery cohort and in two independent validation cohorts.

On top of the heatmap for the AUMC cohort silhouette width score, isopureR score, cluster membership and for the TCGA cohort silhouette width score, isopureR score, number of SNP mutations, cluster membership are indicated.

## 4. Discussion

Based on the unpredictable response to therapy and the large inter-patient variation in survival outcomes, clearly EAC is a heterogeneous disease [12]. Here, for the first time via unsupervised hierarchical clustering on RNA-seq profiles, we describe the existence of three well-defined subgroups in EACs. The results are primarily based on the analysis of >100 transcriptomic profiles from high quality RNA obtained from treatment-naïve patient biopsies. The robustness of these three subgroups was confirmed in a set of EAC profiles from the TCGA database. Moreover, by using a smaller set of differentially expressed genes and identifying a smaller number of key signaling pathways by using the Nanostring PanCancer progression panel, we demonstrated that these subgroups can be efficiently identified in FFPE samples. We feel that Nanostring technology on FFPE should be considered as a basic tool to subclassify EACs in order to gain insight into tumor type, which can aid in tailoring therapy and clinical decision-making. Indeed, these subgroups were associated with distinct transcriptomic, histopathological and clinical characteristics and response to neo-adjuvant therapy.

In this study, we have chosen to use bulk tumor samples containing both the epithelial tumor cell compartment next to the stromal and infiltrating cells. Therefore, the quantified transcriptomes reflect a complexity of profiles rather than purified epithelial cancer cell signatures. It has been demonstrated that both the expression signatures by stromal and immunologic/infiltrating cells are important for tumor behavior and have prognostic value [9]. The crosstalk between cancer cells and their microenvironment is crucial for oncogenic processes such as metastatic progression [20].

In the current study, patients from subgroup two were characterized by activated immune pathways, and in contrast, EAC subgroup three had a tendency for better survival compared to subgroups one and two, and showed de-activation of cell adhesion and immune pathways. This is in line with other cancer types of the gastro-intestinal tract in which prognostic “stromal” subgroups have been identified. For colorectal cancer, the mesenchymal-activated subgroup had poorer outcomes than the three other subgroups [21]. In pancreatic cancer, a “normal” versus a more aggressive “activated stromal” subgroup was identified [9]. Whether the deactivation of stromal cells in subgroup three is the result of a less active stromal tumor environment with lower gene expression levels, or due to a lower number of epithelial cells undergoing Epithelial Mesenchymal Transition, remains uncertain.

The second objective of this study was to evaluate the gene expression signatures for potential associations with response to neo-adjuvant therapy. Mandard score and pT are classifications which previously have been associated with survival of EAC (treated with cisplatin-based chemotherapy) [12,22]. Our results showed that the highest number of differentially expressed genes was seen between complete versus incomplete responders, indicating biological differences between these two groups. Moreover, cases from subgroup one, who were treated with neo-adjuvant therapies, proved to be more therapy-resistant as indicated by the higher pT in the resection specimens, which may indicate that under certain circumstances these patients could benefit more from alternative (neo-) adjuvant strategies or from surgery without (current) neo-adjuvant treatment. This subgroup is characterized by activated p38 MAPK and activated Toll-like receptor signaling [23,24]. In general, p38 MAPK pathways are activated by cellular stress and are associated with growth regulating signaling [25]. It has been demonstrated that activation of p38 MAPK pathways in EAC is associated with less apoptosis and increased cell proliferation [26].

Because of poor survival rates, the potential of adding immunotherapy to improve EAC outcomes is a topic of great interest. In several trials, resistant and metastatic cases were selected based on the immune infiltrates of the cancers. In our analysis, patients treated with neo-adjuvant therapies from our discovery cohort showed differences in the immune phenotypes. Therefore, we used CIBERSORTx cell sorting technology to analyze the RNA sequencing data to compare the cellular composition of the tissues of complete responders to that of poor responders. In another study, CIBERSORTx showed that a subset of patients with advanced solid cancers, characterized by early-on increase of T follicular helper cells after treatment with pembrolizumab immunotherapy, had a favorable progression-free survival [27]. The main differences that we found were more activated CD4 memory T-cells and neutrophils in responders. When we compared our results to similar material of two smaller independent cohorts of patients treated with nCRT, we found that one of these cohorts had a higher number of both activated and resting dendritic cells in the complete responders. Activated dendritic cells are of importance for the regulation of the innate and adaptive immune system [28]. Immature dendritic cells are able to recognize foreign cytosolic DNA, for example by the cytosolic DNA sensing pathway [29]. The cytosolic DNA sensing pathway was also upregulated in the complete responders. Additionally, the activation of the chemokine and cytokine–cytokine receptor interaction pathway, as seen in complete responders in the discovery cohort, potentially reflects pro-inflammatory cytokine release, for instance, by dendritic cells [30].

One of the most important functions of dendritic cells is to raise anti-tumor cytotoxic T cell responses by cross-presentation of antigens [31]. In our previous work, we have shown that higher expression of MHC class I molecules, which are involved in antigen presentation to cytotoxic T cells, correlated with higher expression of genes that regulate adaptive immune responses (PD-L1, PD-L2, IDO1) and are associated with poor response to nCRT and poor survival in EAC [32]. Besides cytotoxic T-cells, B-cells are also involved in the adaptive immune response. The complete responders in the discovery cohort had a low number of activated antibody-producing B-cells (plasma cells). The low number of B-cells was also observed in the responders of one of the smaller (n = 22) independent validation cohorts. The presence of the different types of immune cells in EACs is of interest with respect to response to the current immunotherapies which are offered to EAC patients as adjuvant therapy, or, in the case of metastatic disease, within several trials [33,34,35].

## 5. Conclusions

Our study was exploratory in nature, with relatively small cohorts for investigating response to CRT signatures and independent validation, but importantly contributes to the current knowledge regarding molecular heterogeneity and therapy responsiveness in EACs. In summary, we report on the existence of three distinct molecular subgroups in EAC based on gene expression. This classification contributes to our knowledge on the specific molecular backgrounds of EACs. Moreover, the differences in gene expression between responders and non-responders support a biological foundation underlying response to chemo-and chemoradiotherapy. Differential expression and pathway analysis indicate promising targets for immune therapy in specific subgroups. Moreover, we believe that based on our findings, gene expression profiling of EACs, for instance by using Nanostring technology (Pan-Cancer progression panel) on FFPE samples, should be applied to subclassify EAC cases in order to select the most appropriate therapy for the individual patient.

## Figures and Tables

**Table 1:** Baseline characteristics of AUMC (discovery cohort) and TCGA database.

Characteristics	AUMC (n = 107)	TCGA (n = 77)	*p* Value
Sex			
Male/female (no.)	95/12	66/11	
Male/female (%)	89/11	86/14	0.65 †
Age			
Median (IQR) years	66.7 (15.7) years	68.0 (19.0) years	0.65 ^
Year of diagnosis			-
’98/’99/’00/’01/’04/’05/’06/’07/’08/’09/’10/	0/0/0/0/0/0/0/0/0/0/0/	1/2/8/11/5/3/5/2/1/3/8/	
’11/‘12/’13/’14/’15/’16/’17/NA (no.)	0/16/20/16/27/22/6/0	5/12/6/0/0/0/0/5	
AJCC Stage			0.00 **
I/II/III/IV/NA (no.)	0/27/65/12/3	10/22/32/10/3	
I/II/III/IV (%)	0/26/63/12	14/30/43/14	
Clinical T stage at baseline			-
Tx/T0/T1/T2/T3/T4/NA (no.)	4/0/2/19/78/4/0	2/0/1/2/13/59	
Tx/T0/T1/T2/T3/T4 (%)	4/0/2/18/73/4	-	
Clinical T stage at baseline in patients treated with nC(R)T/surgery with or without adjuvant therapy		NA	NA
Tx/T0/T1/T2/T3/T4/NA (no.)	3/0/1/11/51/2/0		
Tx/T0/T1/T2/T3/T4 (%)	4/0/1/16/75/3		
Histologic grade			0.00 *
G1/G2/G3/NA (no.)	23/33/46/5	1/27/24/25	
G1/G2/G3 (%)	23/32/45	2/52/46	
Lauren classification		NA	NA
Intestinal/Diffuse/Mixed/NA (no.)	70/14/22/1		
Intestinal/Diffuse/Mixed (%)	66/13/21		
Signet ring cells		NA	NA
present/absent/NA (no.)	25/56/26		
present/absent (%)	31/69		
Patients per initial treatment strategy n(%)If applicable also Mandard (m) scorem1/m2/m3/m4/m5/NA (no.)			
*nCRT CROSS* */surgery*	46(43%) 6/9/17/12/0/2	0	-
*nCRT CROSS/surgery/adjuvant SOX*	10 (9%) 3/5/2/0/0/0	0	-
*nCRT CROSS+TRAP/surgery*	4 (4%) 1/1/2/0/0/0	0	-
*nCRT CROSS/no surgery*	7 (7%)	0	-
*nCT EOX* */surgery/adjuvant EOX*	4 (4%) 2/0/0/0/1/1	0	-
*nCT EOX/surgery*	3 (3%) 0/1/0/2/0/0	0	-
*nCT ECC/surgery/adjuvant ECC*	1 (1%) 0/0/0/1/0/0	0	-
*only surgery*	1 (1%)	0	-
*dCRT carboplatin/paclitaxel/no surgery*	16 (15%)	0	-
*palliative therapy*	13 (12%)	0	-
*No treatment*	2 (2%)	0	-
*Surgery, priorly treated with radiation and chemotherapy (but no neo-adjuvant therapy)*	0	2 (3%)	-
*No neo-adjuvant therapy, surgery*	0	65 (84%)	-
*No neo-adjuvant therapy, no surgery*	0	10 (13%)	-
Mandard score in all patients treated with nC(R)T/surgery with or without adjuvant therapy (n = 70)		NA	NA
1/2/3/4/5/NA (no.)	12/16/21/15/1/3		
1/2/3/4/5 (%)	18/25/32/23/2		
T stage resection specimen in all patients treated with nC(R)T/surgery with or without adjuvant therapy (n = 70)		NA	NA
Tx/T1/T2/T3/T4/NA (no.)	12/14/12/26/2/2		
Tx/T1/T2/T3/T4 (%)	18/21/18/39/3		

† Fisher’s exact test two-sided; ^ Mann-Whitney-Wilcoxon test (Age is not normally distributed according to QQ-plot and histogram); * Chi square test; ** Chi square test with simulated *p*-value (2000 replicates); Number (no.); InterQuartile Range (IQR); Not Available (NA); neo-adjuvant Chemo(Radio)Therapy (nC(R)T); neo-adjuvant ChemoRadioTherapy with carboplatin and paclitaxel *+ 41.4 Gy* according to the CROSS trial (nCRT CROSS); adjuvant therapy with S-1 and oxaliplatin (adjuvant SOX); neo-adjuvant trastuzumab and pertuzumab in HER2 positive EAC (TRAP); neo-adjuvant chemotherapy with epirubicin, oxaliplatin and capecitabin (nCT EOX); adjuvant chemotherapy with epirubicin, oxaliplatin and capecitabin (adjuvant EOX); adjuvant chemotherapy with epirubicin, cisplatin and capecitabin (adjuvant ECC); definitive ChemoRadioTherapy with carboplatin and paclitaxel *+ 41.4 Gy* (dCRT carbo/pac). TCGA AJCC Stage derived from Neoplasm.Disease.Stage.American.Joint.Committee.on.Cancer.Code (equal to clinical stage in biolinks file) and Neoplasm.Disease.Stage.American.Joint.Committee.on.Cancer.Code.1 (equal to pathological stage in biolinks file) from Cbioportal file. TCGA Clinical T stage at baseline derived from stage_event_tnm_categories and stage_event_clinical_stage in biolinks file.

**Table 2:** Differences between subgroups in AUMC database.

Characteristics	AUMC1 (n = 30)	AUMC2 (n = 40)	AUMC3 (n = 37)	*p* Value
Sex				1.00 †
Male/female (no.)	27/3	35/5	33/4	
Male/female (%)	90/9	88/13	89/11	
Age				0.47 ‡
Median (IQR) years	69 (18.1)	66 (14.6)	64(14.0)	
AJCC Stage				0.80 ^^
I/II/III/IV/NA (no.)	0/8/17/5/0	0/8/27/3/2	0/11/21/4/1	
I/II/III/IV (%)	0/27/57/17	0/21/71/8	0/31/58/11	
Clinical T stage at baseline				0.15 ^^
Tx/T1/T2/T3/T4/NA (no.)	0/0/0/4/24/2/0	3/0/0/6/31/0/0	1/0/2/9/23/2/0	
Tx/T1/T2/T3/T4 (%)	0/0/0/13/80/7	8/0/0/15/78/0	3/0/5/24/62/5	
Clinical T stage at baseline in all patients treated with nC(R)T/surgery with or without adjuvant therapy				0.25 *
Tx/T1/T2/T3/T4/NA (no.)	0/0/0/0/16/1/0	2/0/0/5/21/0/0	1/0/1/6/14/1/0	
Tx/T1/T2/T3/T4 (%)	0/0/0/0/94/6	7/0/0/18/75/0	4/0/4/26/61/4	
**Histologic grade**				0.046 *
**G1/G2/G3/NA (no.)**	**4/11/13/2**	**5/13/21/1**	**14/9/12/2**	
**G1/G2/G3 (%)**	**14/39/46**	**13/33/54**	**40/26/34**	
Lauren classification				0.79 *
Intestinal/Diffuse/Mixed/NA (no.)	17/5/8/0	28/5/7/0	25/4/7/1	
Intestinal/Diffuse/Mixed (%)	57/17/27	70/13/18	69/11/19	
Signet ring cells				0.08 †
present/absent/NA (no.)	11/11/8	8/22/10	6/23/8	
present/absent (%)	50/50	27/73	21/79	
Mandard score in all patients treated with nC(R)T/surgery with or without adjuvant				0.18 *
1/2/3/4/5/NA (no.)	3/4/3/5/1/1	8/7/7/5/0/1	1/5/11/5/0/1	
1/2/3/4/5 (%)	19/25/19/31/6	30/26/26/19/0	5/23/50/23/0	
**T resection specimen** in all patients treated with nC(R)T/surgery with or without adjuvant				0.01 *
**Tx/T1/T2/T3/T4/NA (no.)**	**3/1/0/11/2/0**	**7/5/7/7/0/1**	**1/8/5/8/0/1**	
**Tx/T1/T2/T3/T4 (%)**	**18/6/0/65/12**	**30/19/26/26/0**	**5/36/23/36/0**	

† Fisher’s exact test, two-sided; ‡ Kruskal Wallis test (Age is not normally distributed according to QQ-plot and histogram); * Chi-square test; ^^ Asymptotic Generalized Pearson Chi-Squared Test (ordered nominal variable by one non-ordered nominal variable); Number (no.); InterQuartile Range (IQR); Not Available (NA); neo-adjuvant Chemo (Radio)Therapy (nC(R)T).

## Data Availability

RNA sequencing profiles from the discovery cohort and nanostring profiles from the validation cohort will be made accessible at GEO data repository GSE207527.

## Supplementary Materials

The following supporting information can be downloaded at: https://www.mdpi.com/article/10.3390/cancers14184498/s1, Table S1 Differences between subgroups TCGA cohort. Table S2 Baseline characteristics Nanostring cohort. Table S3 Differences between subgroups nanostring cohort. Figure S1. Cluster algorithm in 107 patients with EAC leads to 3 distinct subgroups. Figure S1A. Heatmaps of consensus matrix for k = 3. The consensus matrix have RNA sequencing profiles as both rows and columns. The consensus values range from 0 to1 (respectively never clustered together and always clustered together) marked by white to dark blue. The dendrogram at the top of the heatmap shows the membership of samples to cluster 1, 2 or 3, as a result of consensus clustering. Figure S1B. Consensus Cumulative Distribution Function (CDF) Plot indicating the cumulative distribution functions of the consensus matrix for each k, estimated by a histogram of 100 bins. Figure S1C. Delta Area plot indicating relative change in area under the CDF curve. Figure S1D. PCA plot The subgroups can be recognized on the PCA plot. Figure S2. PCA plot Random forest model leads to identification of similar distinct subgroups in 77 patient EAC profiles from the TCGA cohort. These subgroups can be recognized on the PCA plot. Figure S3. Kaplan Meier analyse. Figure S3A. Overall survival in AUMC cohort. Figure S3B. Overall survival in TCGA cohort. Figure S4. Heatmap of genes from Nanostring PanCancer Progression panel in RNA seq AUMC discovery cohort. Figure S5. Validation of clusters in nanostring cohort. Figure S5A. Heatmap indicating gene expression as quantified by nanostring profiling. Figure S5B. PCA plot. The subgroups can be recognized on the PCA plot. Figure S6: immune cells CIBERSORTx in subgroups AMC and TCGA. Figure S7: clinical T stage and pathological T stage after neoadjuvant treatment according to CROSS in 3 clusters in AUMC cohort. Figure S8A: MA plots indicating results of supervised investigation by differential expression analyses in 54 patients treated with nCRT according to CROSS regimen (followed by surgical resection and treated with/without adjuvant therapy) from the AUMC discovery cohort. Differentially expressed genes in red between patients with Mandard 1 versus higher Mandard scores (left upper plot) and pTx versus higher pT stadia (left lower plot). Figure S8B: Heatmap indicating GSEA results for 54 patients treated with nCRT according to the CROSS regimen (followed by surgical resection and treated with/without adjuvant therapy) from the AUMC cohort. In red enriched KEGG pathways in patients with Mandard 1 and in blue activated KEGG pathways in patients with higher Mandard scores.; File S1 materials and methods. References [36,37,38,39] are cited in the Supplementary Materials.
