# Supplementary material for: Identification of Novel Molecular Subgroups in Esophageal Adenocarcinoma to Predict Response to Neo-Adjuvant Therapies
Source: Cancers (Basel). 2022 Sep 16;14(18):4498. doi: 10.3390/cancers14184498 (PMC9496882; doi:10.3390/cancers14184498)
Supplement: Supplementary file 1 [file cancers-14-04498-s001.zip › Supplementary Materials and Methods.pdf]

Supplementary Files S1

Supplementary Materials and Methods

RNA preparation discovery cohort

RNA was extracted from 110 tumor and 10 normal tissue biopsies with the RNeasy Universal Kit/RNeasy Plus Mini Kit/AllPrep DNA/RNA/miRNA Universal kit (Qiagen, United States, Cat. No. / ID: 73404, Cat. No. / ID: 74134/, Cat. No. / ID: 80224), according to manufacturer's protocol. From every sample, 20 µl of isolated RNA with a concentration between 50-100ng/ul was send to GenomeScan B.V. Leiden, The Netherlands, for further processing.
Sample preparation was performed using the NEBNext Ultra Directional RNA library Prep Kit for Illumina, according to manufacturer's protocol (NEB #E7420S/L). Briefly, mRNA was isolated from total RNA using the oligo-dT magnetic beads. After fragmentation of the mRNA, a cDNA synthesis was performed. This was used for ligation with the sequencing adapters and PCR amplification of the resulting product. To assess the quality and yield after sample preparation, the concentration of the RNA was determined using the Fragment Analyzer. All samples met the quality requirements. The size of the resulting products was consistent with the expected size distribution (a broad peek between 300-500 bp).

RNA seq expression profiling discovery cohort

For the discovery dataset, clustering and cDNA sequencing using the Illumina NextSeq500 was performed according to manufacturer's protocols. A concentration of 1.5-1.6pM of cDNA was used. NextSeq control software v2.0.2 was used.

Image analysis, base calling, and quality check was performed with the Illumina data analysis pipeline RTA v2.4.11 and Bcl2fastq v2.17. Reads were mapped to the current Genome Reference Consortium Human genome build 38 patch release 7 (GRCh38.p7) available via GenCode. Alignment was performed using Tophat2 with default parameters.

Based on the mapped read locations in the alignment file, the frequency of how often a read was mapped on a transcript was determined using featureCounts (1). The counts were saved to count files.

Bioinformatics analyses

For the discovery cohort, count files served as input into the R language and environment for statistical computing (version 2.15.1; R Foundation for Statistical Computing, Vienna, Austria; http://www.R-project.org/). Normalization using the variance stabilizing transformation (vst) function from DESeq2 (2) and correction for batch effects using ComBat from the SVA package (3) was performed.

The ISOpureR package (4) was used to estimate tumor percentage in the tumor samples, by computational analysis of 110 tumor samples and 10 corresponding healthy squamous tissue samples. Only samples with an estimated tumor percentage above 50% (n=107, range= 51-100%) were included in further cluster analysis.

ConsensusClusterPlus

The number of subsamples was set to 1000, the proportion of items to sample set to 0.99, the proportion of features (genes) to sample set to 1, and the cluster range k=2 to k=6. The optimal number of subtypes was determined by investigation of the delta area plot, with relative change in area under the cumulative distribution functions (CDF) curve. Silhouette width was defined by the function silhouette from the cluster package.

Differential expression analyses and Ingenuity Pathway Analysis

Genes that were differentially expressed between subgroups from the discovery cohort were identified by the DESeq function from DESeq2, with cluster membership and batch variables in the design formula. Patients stratified into each of the three subtypes were compared to the patients from the other two subtypes together. Genes with an adjusted p value of <0.05, were considered to be significant differentially expressed.

Enrichment of signaling pathways in the subgroups was investigated by Ingenuity Pathway Analysis (Ingenuity Systems IPA, www.ingenuity.com), Log2 fold change values of differentially expressed genes were used as input. All parameters were default, both direct and indirect gene relationships were considered. Significantly differentially enriched pathways were defined by an adjusted p-value of <0.05 according to the right-tailed Fisher's exact test and a z-score of >=2.0 (predicted activation) or <=-2.0 (predicted deactivation).

Random forest model

Using the discovery dataset, a random forest model was trained on differentially expressed genes. Samples with a positive silhouette width were considered to have a strong association with a given subtype, and were therefore selected to construct the model. By randomly subsetting all classes in the training set to have their class frequencies match the least prevalent class, class imbalances were adjusted for. The number of trees to grow was set to 750 and proximity measure among rows were calculated.

Visualization of differentially expressed genes and significantly enriched pathways

Differentially expressed genes identified in the discovery cohort were plotted in two heatmaps indicating expression in the discovery dataset and in the TCGA dataset. In the heatmap, the rows depict genes and the columns depict different samples. For each sample, the silhouette width and isopureR scores were plotted above the heatmap.

Significantly differentially enriched pathways from the discovery cohort that met the same requirements in the TCGA dataset, were plotted in a heatmap displaying the z-scores calculated for every subgroup by the IPA tool.

Mutation data from the TCGA dataset

Mutation data detected by muse and Copy Number Variation (CNV) data from the TCGA dataset were downloaded. Fisher Exact tests, with adjustment of p-value for multiple comparisons according to Benjamini & Hochberg, were performed to identify genes with CNV and genes with Single-Nucleotide Variant (SNV) mutations that had significant different

incidences between the subgroups. Boxplots indicating the total number of SNVs for each sample were plotted.

NKI/Erasmus MC RNA seq validation cohort for response to neoadjuvant treatment signature

For a validation cohort of the supervised analyses, 51 fresh frozen pre-treatment tumor biopsies from patients with EAC treated with nCRT according to the CROSS regimen followed by surgery, were selected from the Nederlands Kanker Instituut/Netherlands Cancer Institute (NKI) (n=29, Mandard 1 n=5, Mandard 2 n=6, Mandard 3 n=8, Mandard 4 n=9 and Mandard 5 n=1) and the Erasmus MC (n=9, Mandard 1 n=9, Mandard 3 n=5, Mandard 4 n=8).
For the NKI cohort, only biopsies with >70% of tumor content were selected. For the NKI cohort, the AllPrep DNA/RNA FF kit (Qiagen, United States, Cat. No 80311) was used to isolate RNA. For the Erasmus MC cohort, the RNeasy kit (Qiagen, United States, Cat. No74004) was used to isolate RNA.
Concentrations and purities were measured using Bioanalyzer (Agilent, Santa Clara, United States).

RNA was sequenced by GenomeScan B.V. Leiden, The Netherlands. In short, the NEBNext Ultra II Directional RNA Library Prep Kit for Illumina was used according to protocol to process the samples (NEB #E7760S/L). RRNA was depleted from total RNA using the rRNA depletion kit (NEB# E6310). After fragmentation of the rRNA reduced RNA, a cDNA synthesis was performed. This was used for ligation with the sequencing adapters and PCR amplification of

the resulting product. The quality and yield after sample preparation was measured with the Fragment Analyzer. The size of the resulting products was consistent with the expected size distribution (a broad peak between 300-500 bp). Clustering and DNA sequencing using the NovaSeq6000 was performed according to manufacturer's protocols. A concentration of 1.1 nM of DNA was used. Image analysis, base calling, and quality check was performed with the Illumina data analysis pipeline RTA3.4.4 and Bcl2fastq v2.20. Mapping and quantification of reads was performed similarly to the discovery cohort, as described above.

Differential expression analyses comparing patients with different levels of pathological response to nCRT with CROSS according to Mandard were performed for this cohort.

Supplementary Tables

Supplementary Table S1. Differences between subgroups TCGA cohort.

Characteristics	TCGA1 (n=14)	TCGA2 (n=29)	TCGA3 (n=34)	P value
Sex<br>Male/female (no.)<br>Male/female (%)	12/2<br>86/14	25/4<br>86/14	29/5<br>85/15	1.00†
Age<br>median (IQR) years	60.5 (19.25)	74.0 (19.0)	66.0 (16.75)	0.28‡
AJCC Stage<br>I/II/III/IV/NA (no.)<br>I/II/III/IV (%)	3/2/5/2/2<br>25/17/42/17	0/7/17/4/1<br>25/61/14	7/13/10/4/0<br>21/38/29/12	**0.07****
Clinical T stage at baseline<br>Tx/T1/T2/T3/T4/NA (no.)<br>Tx/T1/T2/T3/T4 (%)	0/0/2/4/0/8<br>-	2/0/0/3/0/24<br>-	0/1/0/6/0/27<br>-	-

Clinical T stage at baseline in all patients treated with nC(R)T/surgery with or without adjuvant<br><br>Tx/T1/T2/T3/T4/NA (no.)<br><br>Tx/T1/T2/T3/T4 (%)	NA	NA	NA	NA
**Histologic grade**<br>**G1/G2/G3/NA (no.)**<br><br>**G1/G2/G3 (%)**	**1/7/1/5**<br>**11/78/11**	**0/3/16/10**<br>**0/16/84**	**0/17/7/10**<br>**0/71/29**	**0.00***
Lauren classification<br><br>Intestinal/Diffuse/Mixed/NA (no.)<br>Intestinal/Diffuse/Mixed (%)	NA	NA	NA	NA
**Signet ring cells**<br><br>**present/absent/NA (no.)**<br>**present/absent (%)**	NA	NA	NA	NA
Mandard score in all patients treated with nC(R)T/surgery with or without adjuvant<br><br>1/2/3/4/5/NA (no.)<br><br>1/2/3/4/5 (%)	NA	NA	NA	NA
**T resection specimen** in all patients treated with nC(R)T/surgery with or without adjuvant therapy<br><br>**Tx/T1/T2/T3/T4/NA (no.)**<br>**Tx/T1/T2/T3/T4 (%)**	NA	NA	NA	NA

† Fisher's exact test two sided

‡ Kruskal Wallis test (Age is not normally distributed according to QQ-plot and histogram)
*Chi square test

** Chi square test with simulated p-value (2000 replicates)

Clinical T stage at baseline derived from stage_event_tnm_categories and stage_event_clinical_stage in biolink file.

Number (no.)

InterQuartile Range (IQR)

Not Available (NA)

neoadjuvant Chemo(Radio)Therapy (nC(R)T)

Supplementary Table S2. Baseline characteristics Nanostring cohort

Characteristics	nanostring (n=56)
Center<br><br>Bologna/AUMC (no.)<br>Bologna/AUMC (%)	30/26<br>54/46
Sex<br><br>Male/female (no.)<br><br>Male/female (%)	44/12<br>79/21
Age<br><br>median (IQR) years	71.0 (12) years
Experiment Year<br>2018/2019 (no.)	31/25
AJCC Stage<br><br>I/II/III/IV/NA (no.)<br>I/II/III/IV (%)	0/18/26/6/6<br>0/32/46/11/11
Histologic grade<br>G1/G2/G3/NA (no.)<br><br>G1/G2/G3 (%)	7/24/21/4<br>13/46/40
Lauren classification<br><br>Intestinal/Diffuse/Mixed/NA (no.)<br>Intestinal/Diffuse/Mixed (%)	45/3/1/7<br>92/6/2
Signet ring cells<br><br>present/absent/NA (no.)<br>present/absent (%)	9/43/4<br><br>17/83
Treatment only surgery	56 (100%)
Number (no.)<br><br>InterQuartile Range (IQR)<br><br>Not Available (NA)	

Supplementary Table S3. Differences between subgroups nanostring cohort

Characteristics	nano1 (n=7)	nano2 (n=20)	nano3 (n=29)	P value
**Center**<br>**Bologna/AUMC (no.)**<br>**Bologna/AUMC (%)**	**7/0**<br>**100/0**	**10/10**<br>**50/50**	**13/16**<br>**45/55**	**0.02†**
Sex<br>Male/female (no.)<br>Male/female (%)	6/1<br>86/14	17/3<br>85/15	21/8<br>72/28	0.59†
Age<br>median (IQR) years	67 (5)	74 (7)	70 (19)	0.93‡
AJCC Stage<br>I/II/III/IV/NA (no.)<br>I/II/III/IV (%)	0/0/4/1/2<br>0/0/80/20	0/5/11/4<br>0/25/55/20	0/13/11/1/4<br>0/52/44/4	0.09*
**Histologic grade**<br>**G1/G2/G3/NA (no.)**<br>**G1/G2/G3 (%)**	**0/3/4/0**<br>**0/43/57**	**2/5/12/1**<br>**11/26/63**	**5/16/5/3**<br>**19/62/19**	**0.03***
Lauren classification<br>Intestinal/Diffuse/Mixed/NA (no.)<br>Intestinal/Diffuse/Mixed (%)	7/0/0/0<br>100/0/0	12/3/1/4<br>75/19/6	26/0/0/3<br>100/0/0	0.06*
Signet ring cells<br>present/absent/NA (no.)<br>present/absent (%)	0/7/0<br>0/100	5/14/1<br>26/74	4/22/3<br>15/85	0.3†

† Fisher's exact test two sided

‡ Kruskal Wallis test (Age is not normally distributed according to QQ-plot and histogram)
*Chi square test

Number (no.)

InterQuartile Range (IQR)

Not Available (NA)

Supplementary Figures

Supplementary Figure S1. Cluster algorithm in 107 patients with EAC leads to 3 distinct subgroups.

Supplementary Figure S1A. Heatmaps of consensus matrix for k = 3.
The consensus matrix have RNA sequencing profiles as both rows and columns. The consensus values range from 0 to1 (respectively never clustered together and always clustered together) marked by white to dark blue. The dendrogram at the top of the heatmap shows the membership of samples to cluster 1, 2 or 3, as a result of consensus clustering.

Supplementary Figure S1B. Consensus Cumulative Distribution Function (CDF) Plot indicating the cumulative distribution functions of the consensus matrix for each k, estimated by a histogram of 100 bins.

Supplementary Figure S1C. Delta Area plot indicating relative change in area under the CDF curve.

Supplementary Figure S1D. PCA plot

The subgroups can be recognized on the PCA plot.

Supplementary Figure S2. PCA plot

Random forest model leads to identification of similar distinct subgroups in 77 patient EAC profiles from the TCGA cohort. These subgroups can be recognized on the PCA plot.

Supplementary Figure S3B. Kaplan Meier analyses

Supplementary Figure S3A. Overall survival in AUMC cohort.

Supplementary Figure S3B. Overall survival in TCGA cohort.

Supplementary Figure S4. Heatmap of genes from Nanostring PanCancer Progression panel in RNA seq AUMC discovery cohort.

Supplementary Figure S5. Validation of clusters in nanostring cohort.

Supplementary Figure S5A. Heatmap indicating gene expression as quantified by nanostring profiling.

Supplementary Figure S5B. PCA plot. The subgroups can be recognized on the PCA plot.

Supplementary Figure S6: immune cells CIBERSORTx in subgroups AMC and TCGA.

Supplementary Figure S7: clinical T stage and pathological T stage after neoadjuvant treatment according to CROSS in 3 clusters in AUMC cohort.

Supplementary Figure S8A: MA plots indicating results of supervised investigation by differential expression analyses in 54 patients treated with nCRT according to CROSS regimen (followed by surgical resection and treated with/without adjuvant therapy) from the AUMC

discovery cohort.

Differentially expressed genes in red between patients with Mandard 1 versus higher Mandard scores (left upper plot) and pTx versus higher pT stadia (left lower plot).

Supplementary Figure S8B:

Heatmap indicating GSEA results for 54 patients treated with nCRT according to the CROSS regimen (followed by surgical resection and treated with/without adjuvant therapy) from the AUMC cohort. In red enriched KEGG pathways in patients with Mandard 1 and in blue activated KEGG pathways in patients with higher Mandard scores.

References

1. Liao Y, Smyth GK, Shi W. featureCounts: an efficient general purpose program for assigning sequence reads to genomic features. Bioinformatics. 2014;30(7):923-30.
2. Love MI, Huber W, Anders S. Moderated estimation of fold change and dispersion for RNA-seq data with DESeq2. Genome Biology. 2014;15(12):550.
3. JT L. sva: Surrogate Variable Analysis. In: WE J, editor. R package version 34202021.
4. Anghel CV, Quon G, Haider S, Nguyen F, Deshwar AG, Morris QD, et al. ISOpureR: an R implementation of a computational purification algorithm of mixed tumour profiles. BMC Bioinformatics. 2015;16:156-.
