# Supplementary figures and images for: Identification of Novel Molecular Subgroups in Esophageal Adenocarcinoma to Predict Response to Neo-Adjuvant Therapies
Source: Cancers (Basel). 2022 Sep 16;14(18):4498. doi: 10.3390/cancers14184498 (PMC9496882; doi:10.3390/cancers14184498)

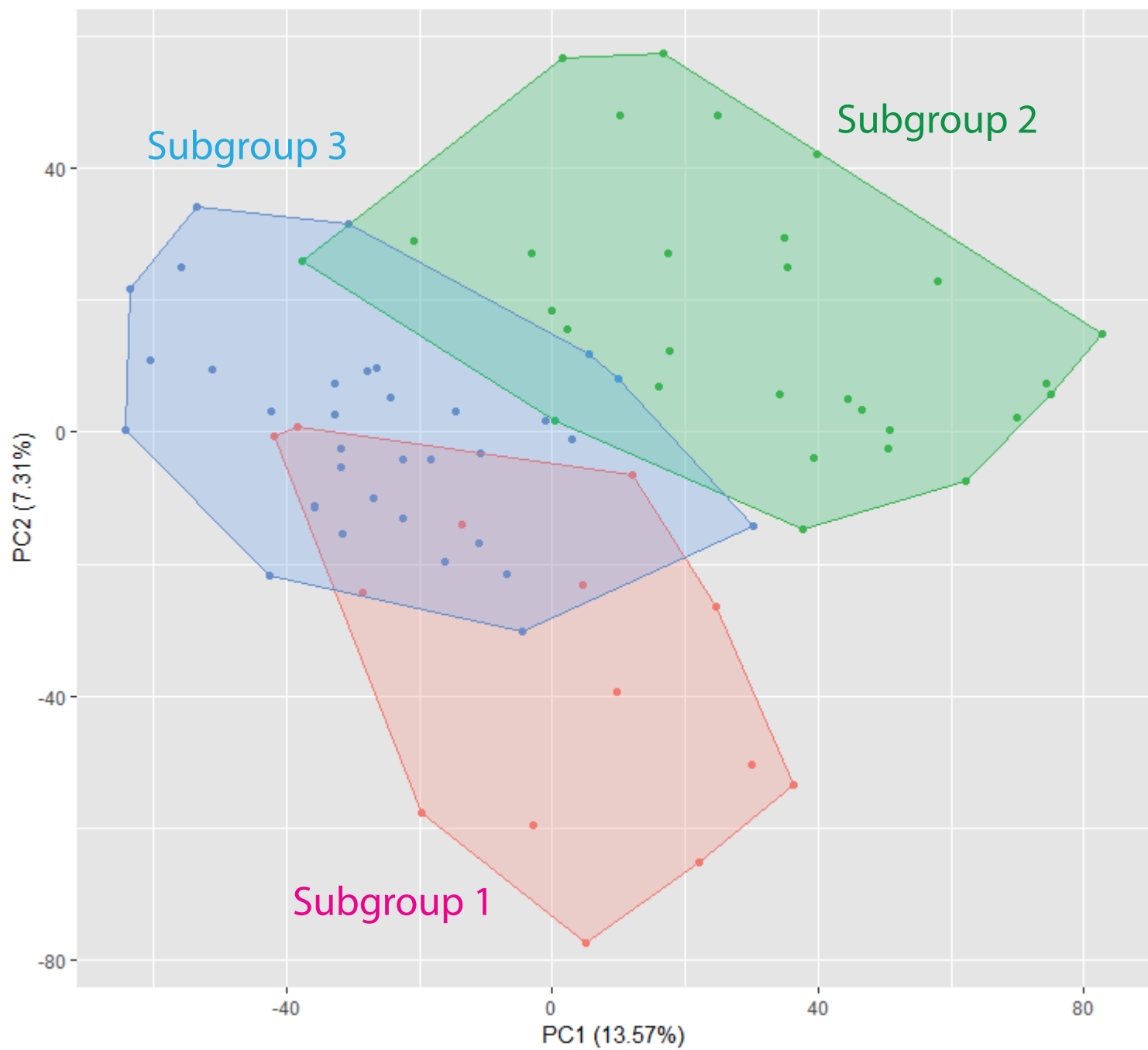

Supplement: Supplementary file 1 [file cancers-14-04498-s001.zip › FigureSupp2.pdf]

A Discovery cohort

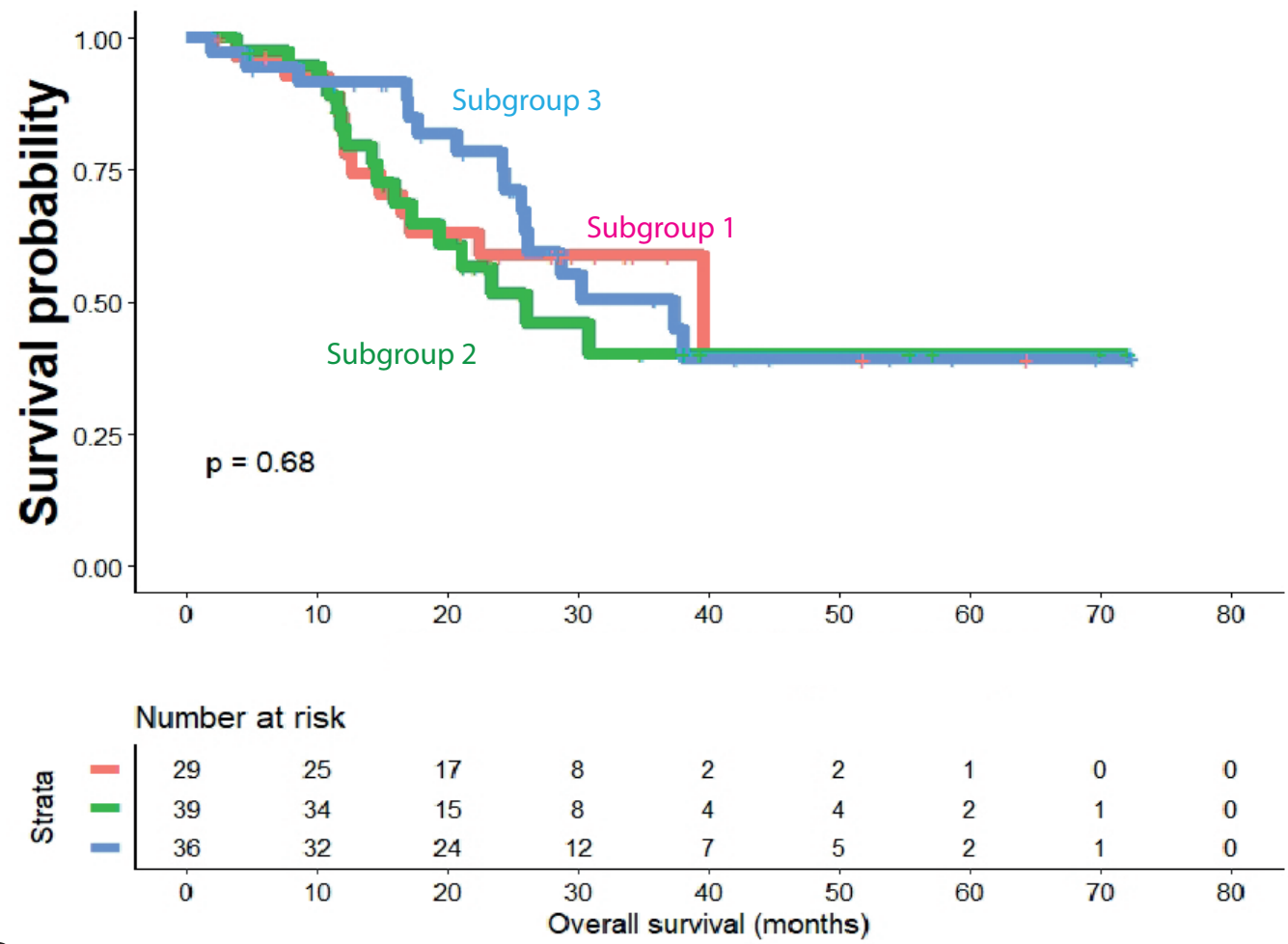

B TCGA

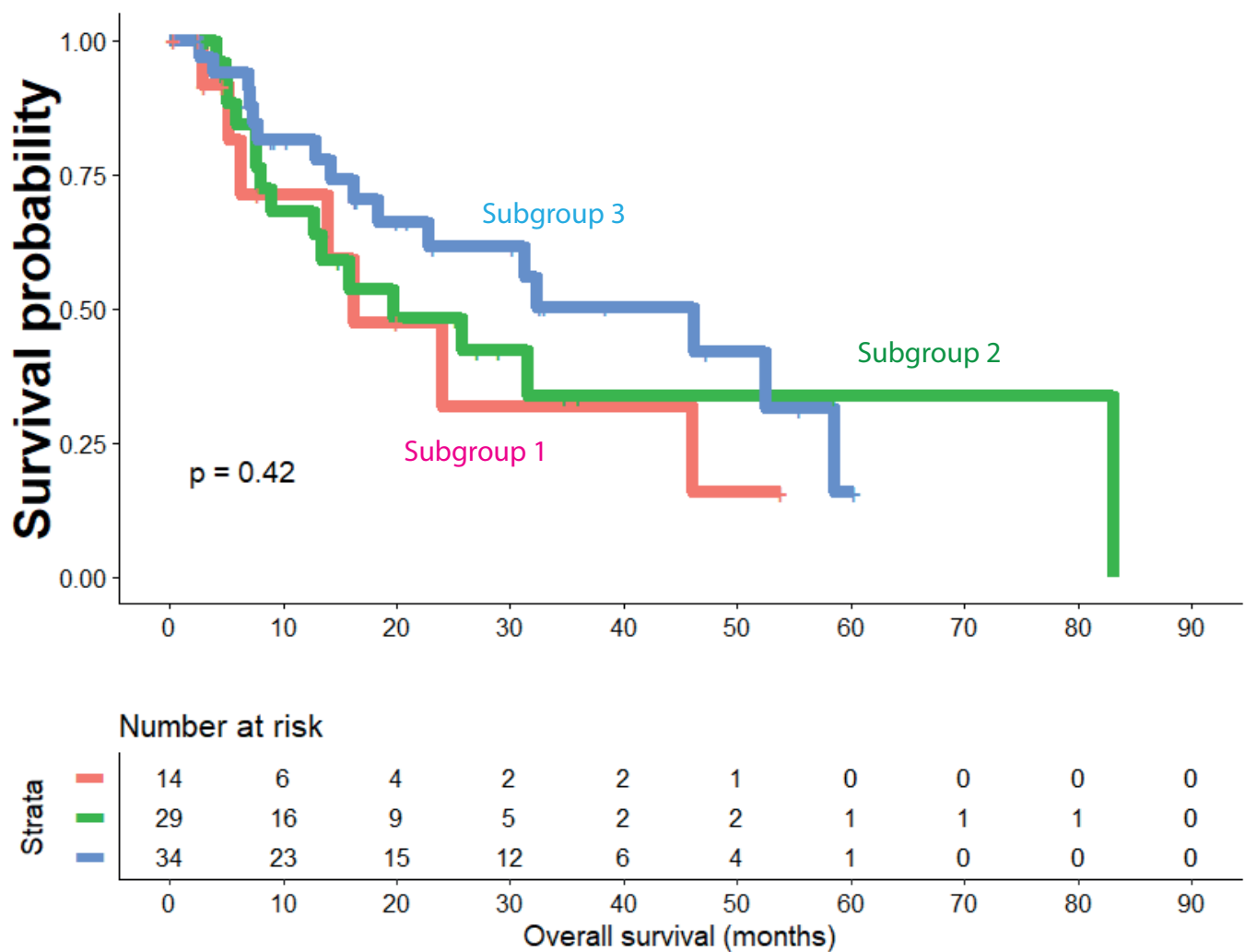

Supplement: Supplementary file 1 [file cancers-14-04498-s001.zip › FigureSupp3.pdf]

Subgroup 1

Subgroup 2

Subgroup 3

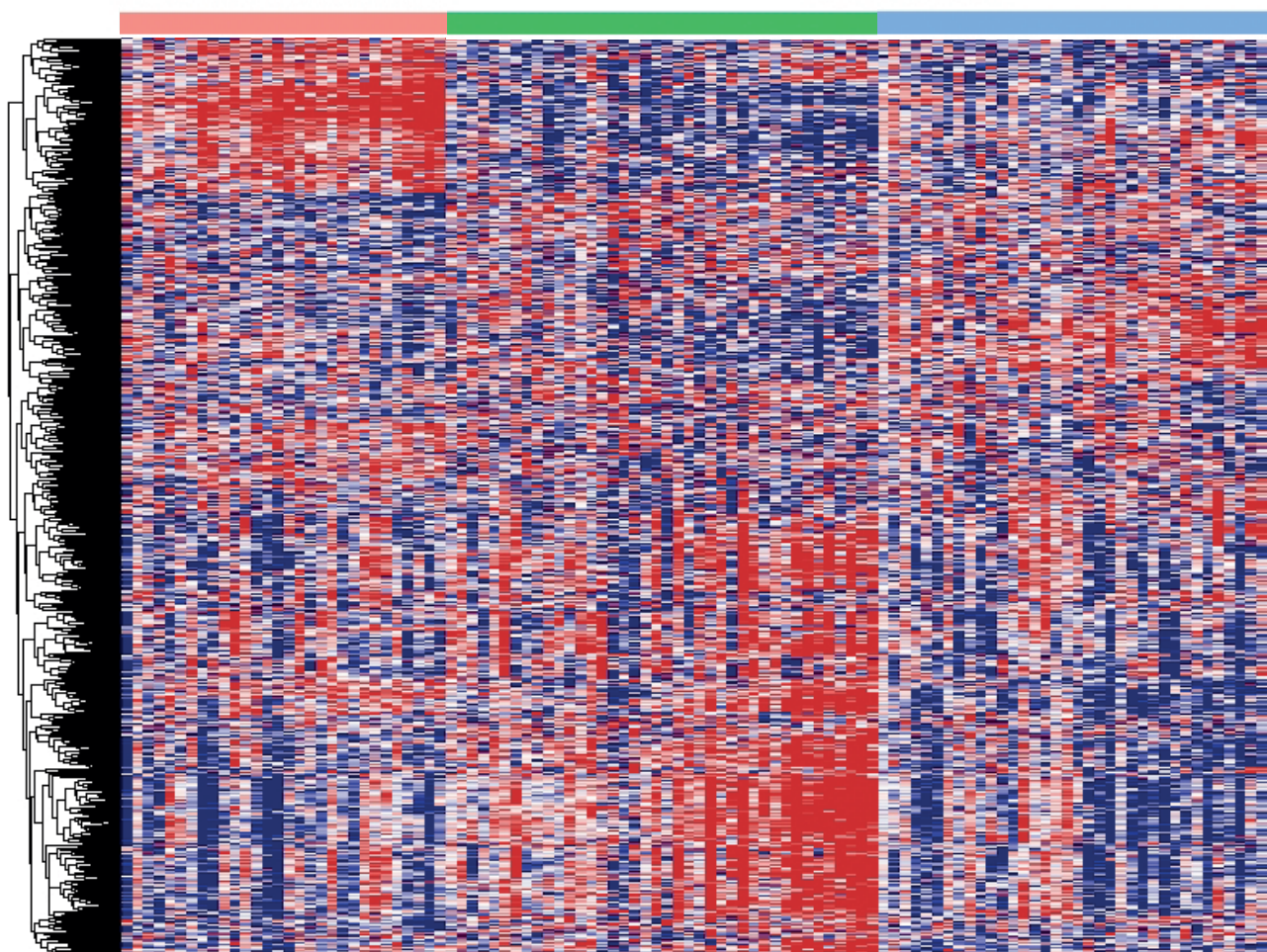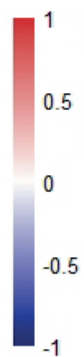

Supplement: Supplementary file 1 [file cancers-14-04498-s001.zip › FigureSupp4.pdf]

A

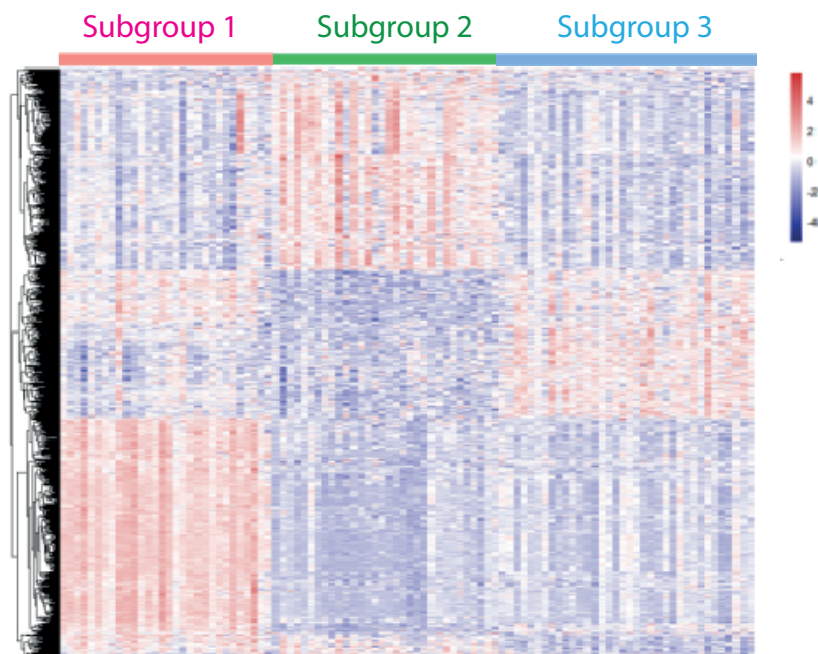

B

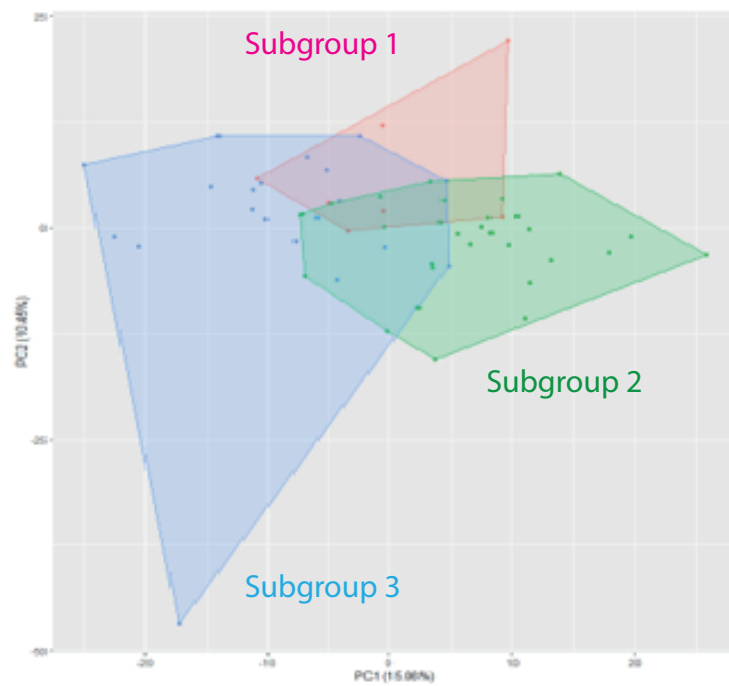

Supplement: Supplementary file 1 [file cancers-14-04498-s001.zip › FigureSupp5.pdf]

## Discovery cohort

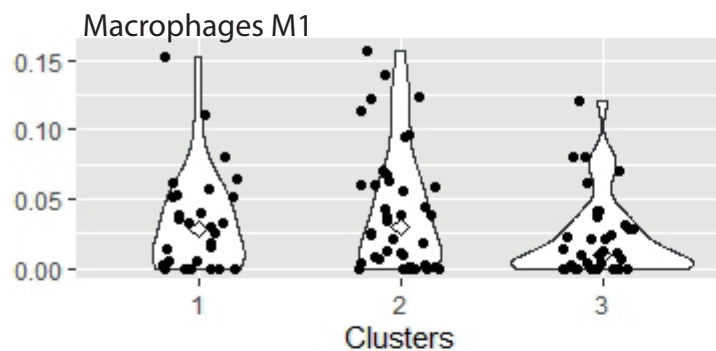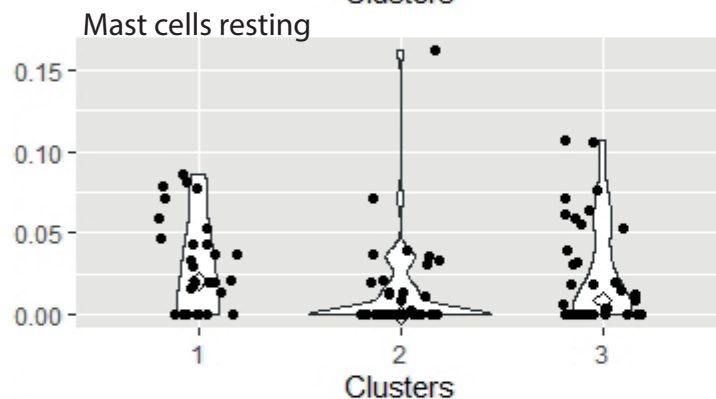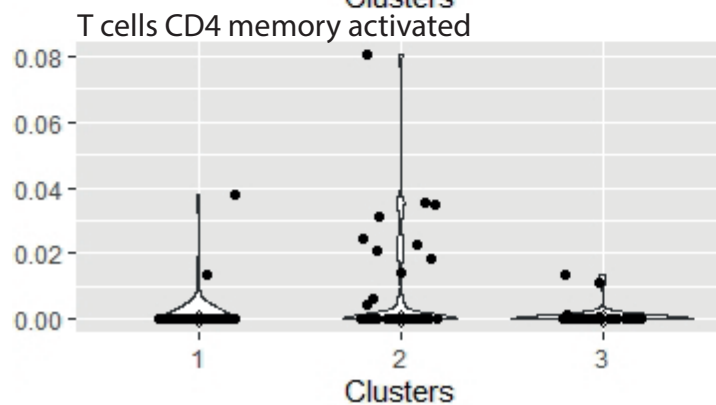

## TCGA

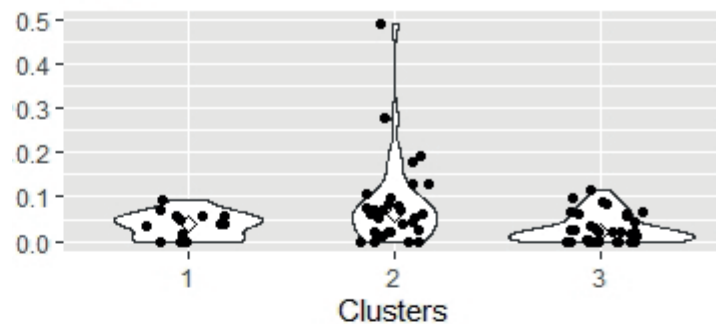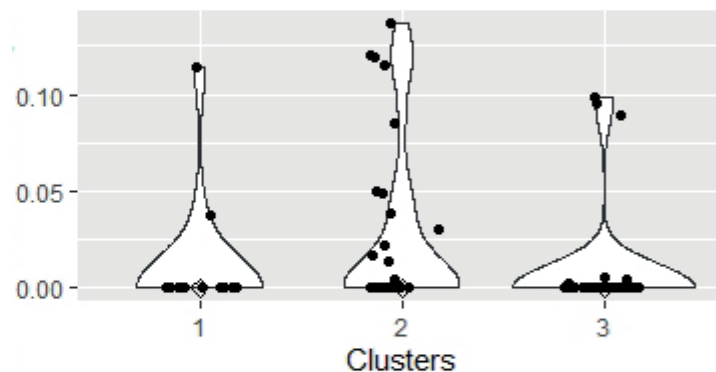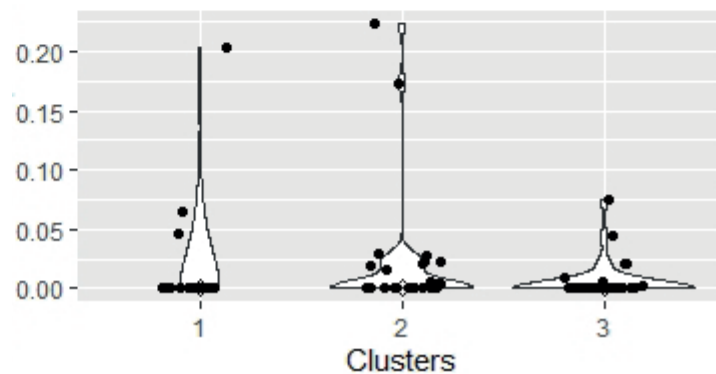

Supplement: Supplementary file 1 [file cancers-14-04498-s001.zip › FigureSupp6.pdf]

Subgroup 1

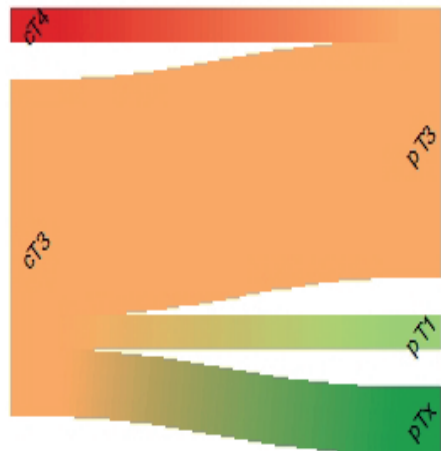

Subgroup 2

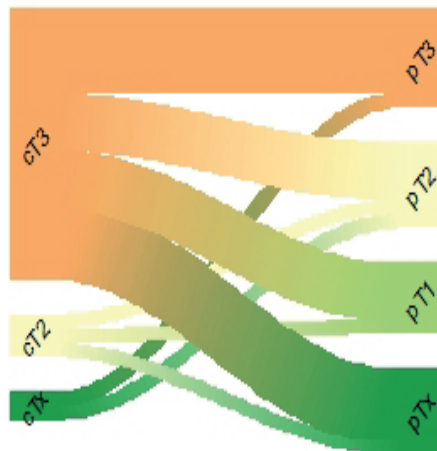

Subgroup 3

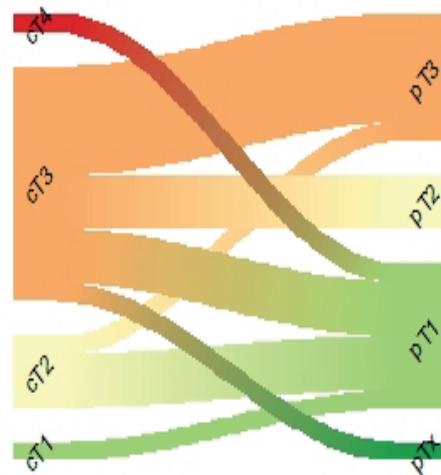

Supplement: Supplementary file 1 [file cancers-14-04498-s001.zip › FigureSupp7.pdf]
