# Supplementary material for: Identification of Novel Molecular Subgroups in Esophageal Adenocarcinoma to Predict Response to Neo-Adjuvant Therapies
Source: Cancers (Basel). 2022 Sep 16;14(18):4498. doi: 10.3390/cancers14184498 (PMC9496882; doi:10.3390/cancers14184498)
Supplement: Supplementary file 1 [file FigureSupp1_rebuttal.pdf › FigureSupp1_rebuttal.pdf]

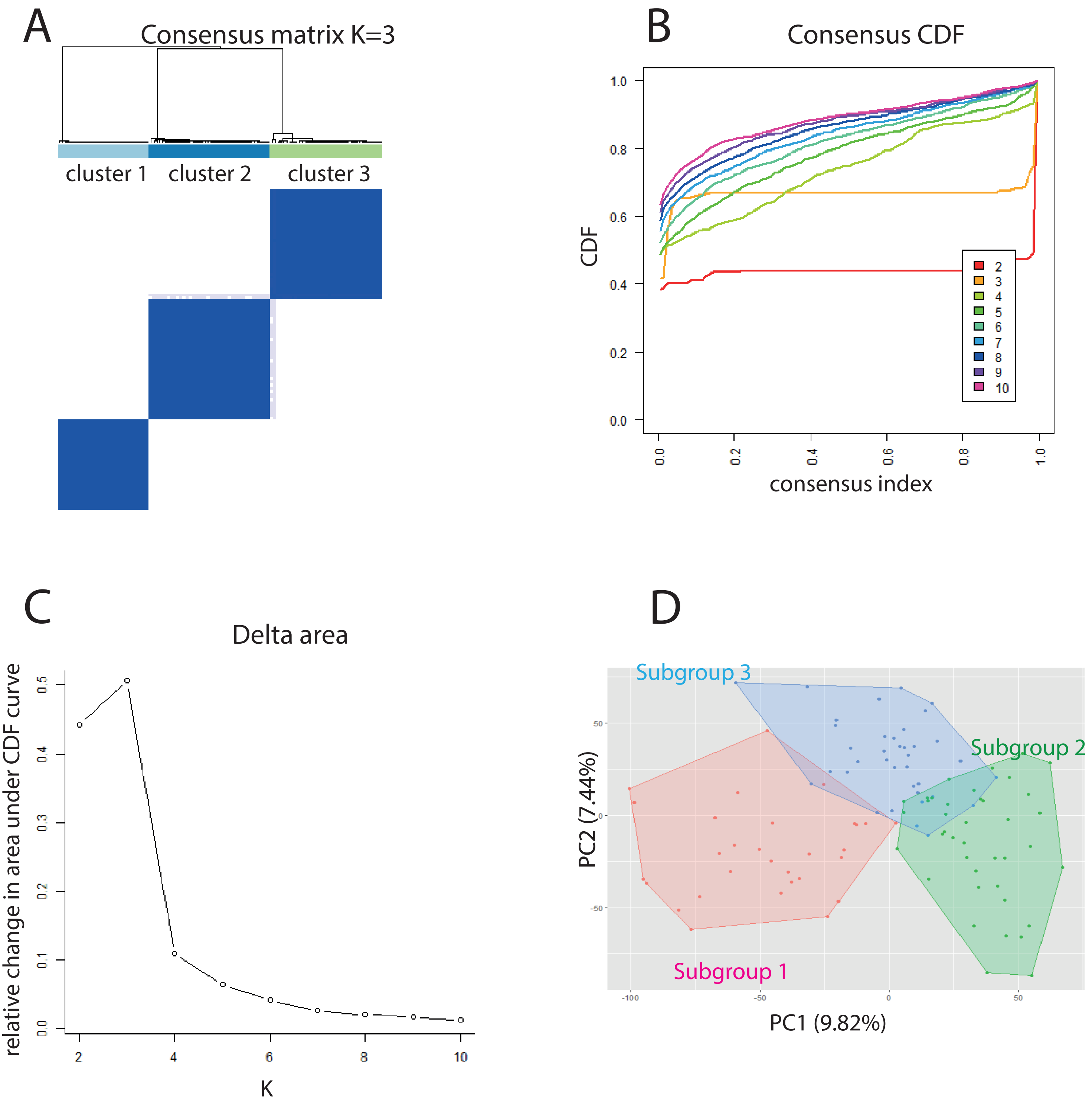
A
Consensus matrix K=3
cluster 1
cluster 2
cluster 3
B
Consensus CDF
CDF
consensus index
1.0
0.8
0.6
0.4
0.2
0.0
2
3
4
5
6
7
8
9
10
C
Delta area
relative change in area under CDF curve
K
0.5
0.4
0.3
0.2
0.1
0.0
2
4
6
8
10
D
Subgroup 3
Subgroup 2
Subgroup 1
PC2 (7.44%)
PC1 (9.82%)
50
0
-50
-100
-50
0
50
