# Supplementary material for: Identification of Novel Molecular Subgroups in Esophageal Adenocarcinoma to Predict Response to Neo-Adjuvant Therapies
Source: Cancers (Basel). 2022 Sep 16;14(18):4498. doi: 10.3390/cancers14184498 (PMC9496882; doi:10.3390/cancers14184498)
Supplement: Supplementary file 1 [file FigureSupp8_rebuttal.pdf › FigureSupp8_rebuttal.pdf]

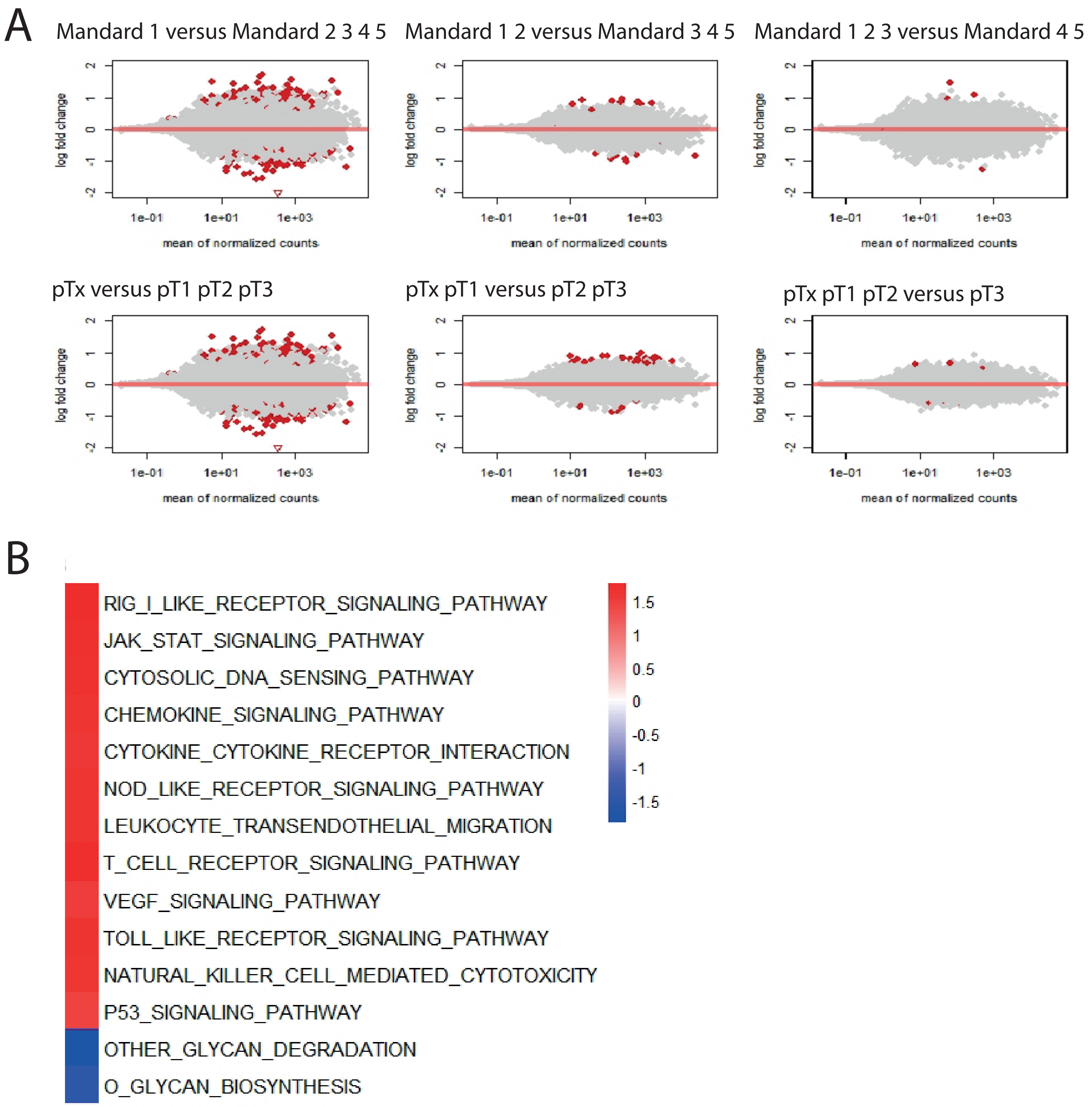
A
Mandard 1 versus Mandard 2 3 4 5
Mandard 1 2 versus Mandard 3 4 5
Mandard 1 2 3 versus Mandard 4 5
pTx versus pT1 pT2 pT3
pTx pT1 versus pT2 pT3
pTx pT1 pT2 versus pT3
log fold change
mean of normalized counts
1e-01
1e+01
1e+03
2
1
0
-1
-2
B
RIG_I_LIKE_RECEPTOR_SIGNALING_PATHWAY
JAK_STAT_SIGNALING_PATHWAY
CYTOSOLIC_DNA_SENSING_PATHWAY
CHEMOKINE_SIGNALING_PATHWAY
CYTOKINE_CYTOKINE_RECEPTOR_INTERACTION
NOD_LIKE_RECEPTOR_SIGNALING_PATHWAY
LEUKOCYTE_TRANSENDOTHELIAL_MIGRATION
T_CELL_RECEPTOR_SIGNALING_PATHWAY
VEGF_SIGNALING_PATHWAY
TOLL_LIKE_RECEPTOR_SIGNALING_PATHWAY
NATURAL_KILLER_CELL_MEDIATED_CYTOTOXICITY
P53_SIGNALING_PATHWAY
OTHER_GLYCAN_DEGRADATION
O_GLYCAN_BIOSYNTHESIS
1.5
1
0.5
0
-0.5
-1
-1.5
